# Association analysis of production traits of Japanese quail (*Coturnix japonica*) using restriction-site associated DNA sequencing

**DOI:** 10.1038/s41598-023-48293-0

**Published:** 2023-12-02

**Authors:** Mohammad Ibrahim Haqani, Michiharu Nakano, Atsushi J. Nagano, Yoshiaki Nakamura, Masaoki Tsudzuki

**Affiliations:** 1 Graduate School of Integrated Sciences for Life, Hiroshima University, Higashi-Hiroshima, Hiroshima, 739-8525 Japan; 2 Faculty of Agriculture and Marine Sciences, Kochi University, Nankoku, Kochi 783-8502 Japan; 3 Faculty of Agriculture, Ryukoku University, Otsu, Shiga 520-2194 Japan; 4 Institute for Advanced Biosciences, Keio University, Yamagata, 997-0017 Japan; 5 Japanese Avian Bioresource Project Research Center, Hiroshima University, Higashi-Hiroshima, Hiroshima, 739-8525 Japan

**Keywords:** Animal breeding, Genetic association study

## Abstract

This study was designed to perform an association analysis and identify SNP markers associated with production traits of Japanese quail using restriction-site-associated DNA sequencing. Weekly body weight data from 805 quail were collected from hatching to 16 weeks of age. A total number of 3990 eggs obtained from 399 female quail were used to assess egg quality traits. Egg-related traits were measured at the beginning of egg production (first stage) and at 12 weeks of age (second stage). Five eggs were analyzed at each stage. Traits, such as egg weight, egg length and short axes, eggshell strength and weight, egg equator thickness, yolk weight, diameter, and colour, albumen weight, age of first egg, total number of laid eggs, and egg production rate, were assessed. A total of 383 SNPs and 1151 associations as well as 734 SNPs and 1442 associations were identified in relation to quail production traits using general linear model (GLM) and mixed linear model (MLM) approaches, respectively. The GLM-identified SNPs were located on chromosomes 1–13, 15, 17–20, 24, 26–28, and Z, underlying phenotypic traits, except for egg and albumen weight at the first stage and yolk yellowness at the second stage. The MLM-identified SNPs were positioned on defined chromosomes associated with phenotypic traits except for the egg long axis at the second stage of egg production. Finally, 35 speculated genes were identified as candidate genes for the targeted traits based on their nearest positions. Our findings provide a deeper understanding and allow a more precise genetic improvement of production traits of Galliformes, particularly in Japanese quail.

## Introduction

Japanese quail is a model bird from the Galliformes order that is raised for production and biological research purposes. The Japanese quail holds significant importance as a bird species owing to its compact size, efficient productivity, quick generational turnover, and early sexual maturity, typically around 6 weeks of age^1^. Quail production traits are important characteristics in the poultry industry, and quantitative trait loci (QTLs) associated with these traits have been identified^2,3^.

Molecular markers have become a key prerequisite for association mapping and are of increasing importance in animal breeding and genetics. Association analysis is an advantageous technology that identifies QTLs underlying phenotypic traits^4^ and provides a link for breeders to select based on genetic information^5^. Among the molecular markers, single nucleotide polymorphism (SNP) markers are widely used^6–9^ due to their abundance in any genome and the cost-efficient identification methods^10^. Genotyping by sequencing (GBS) is a next-generation sequencing (NGS) platform that opens new possibilities for SNP marker identification and can be used in a simple, highly multiplexed system for constructing reduced representation libraries involving inexpensive barcoding, reduced sample handling, no size fractionation, and which requires fewer polymerase chain reaction and purification steps^11^. Restriction-site associated DNA sequencing (RAD-seq)^12–14^ is a GBS that can identify, verify, and score thousands of SNPs simultaneously, reduce complexity across genomes, deliver high-resolution population genomic data, and is convenient for non-model species at a reasonable cost^15^. Existing genotyping platforms represent efficient tools for association studies to increase the output of marker-assisted selection (MAS).

Association analysis is a viable approach for linking phenotypes and genotypes in poultry genetics as well as dissecting complex traits. This type of analysis can boost the information obtained from QTL studies through MAS implementation*.* Quail production traits are complex traits that are controlled by QTLs and can be affected by environmental factors. The QTLs related to production traits of Japanese quail have been previously identified. Reference^2^ identified 22 QTLs for body weight, egg weight, number of eggs laid, and age of the first egg using SNP markers. References^16,17^ discovered four QTLs for body weight and nine QTLs for egg-related traits using the RAD-seq method. Moreover, microsatellite markers have been used to detect QTLs for traits associated with body weight traits^18–23^, growth and egg production^3,24^, and egg-laying curves^25^. Previous studies have also investigated the QTLs underlying features other than production traits of Japanese quail^26–30^. Although molecular breeding helps breeders select the production traits expressed in Japanese quail, association analysis studies in poultry have mostly focused on chickens rather than Japanese quail. In recent years, genome-wide association studies (GWASs) have been widely used in studies of different chicken breeds using a high-density genotyping platform to identify SNPs and candidate genes associated with production traits^31–37^. Numerous studies used GWASs to identify SNPs that control the candidate genes responsible for body weight traits of chickens^38–41^. Similar studies have been conducted for identifying SNPs associated with chicken egg-related traits^42–44^. References^45–48^ performed GWASs for body composition and meat quality traits of chickens.

Studies of association analysis in Japanese quail are limited and mainly concentrated on the association between polymorphisms of gonadotropin-releasing hormone genes and growth traits^49^, prolactin receptor genes and growth traits^50^, and gut microbial architecture of efficiency traits^51^. Existing reports on the genetic map of Japanese quail have been integrated and aligned with assembled chicken sequence data^52^. As Japanese quail shows close phylogenetic relatedness to chickens, having a similar genome length (1.2 × 10^9^ base pairs), chromosome number (2n = 78), and homology of chromosome morphology, a high rate of synteny conservation is expected between the two species^53^. In addition to studies on the quail genome^54,55^, reports on the chicken genome will help breeders investigate genetic analysis and identify SNP markers associated with the targeted traits within the poultry industry. However, to date, there are no published reports on association studies of production traits of Japanese quail using GLM and MLM models with RAD-seq data. Therefore, a study pursuing the identification of SNP markers associated with production traits will provide breeders with a useful tool to assist in selecting high-production lines, specifically in Japanese quail breeding programs and poultry in general. Here, we performed an association analysis and identified SNP markers associated with production traits of Japanese quail using the RAD-seq method. We expect these results to help improve poultry breeding programs and increase production levels through molecular breeding using SNP markers with MAS.

## Materials and methods

### Experimental design

Animal care, experimental protocols, and blood sample collections were approved and conducted in accordance with the Rules on Experimental Animals and Animal Experiments at Hiroshima University, Graduate School of Integrated Sciences for Life, Laboratory of Animal Breeding and Genetics (Approval No. C20-15) and the protocol described in the Guidelines for Proper Conduct of Animal Experiments, Science Council, Japan. https://www.scj.go.jp/ja/info/kohyo/pdf/kohyo-20-k16-2e.pdf. Also, we confirm that all methods have been conducted in adherence to the ARRIVE guidelines (https://arriveguidelines.org). Experimental birds were reared at the Research Farm of Hiroshima University, Japan. Large- and normal-sized (LS and NS) Japanese quail strains were selected as parental strains. The LS Japanese quail is known for its high body weight, reaching around 200 g in females and 170 g in males upon maturity, making it suitable for meat production purposes. Conversely, the NS strain of Japanese quail exhibits a normal body weight, approximately 130 g in females and 100 g in males at the age of maturity, and has been primarily used for egg production^1^. These two strains originate from genetically distinct backgrounds concerning the specific traits of interest, which could potentially aid in accurately identifying targeted SNPs. One hundred quails from each of the two strains, consisting of 50% males and 50% females, were reared as parents. The cross involved an LS male paired with three NS females, and an NS male paired with three LS females, resulting in 100 F_1_ progeny in a reciprocal cross (50 males and 50 females). Subsequently, a total of 505 F_2_ birds (256 males and 249 females) were generated from a reciprocal cross between six F_1_ males and 18 F_1_ females. Newly hatched chicks were instantly pedigree leg-banded and weighed before being moved to heated brooders, where they were reared until four weeks of age. Thereafter, quail were housed in individual steel wire meshed cages (15 cm deep, 18 cm wide, and 18 cm high) equipped with feeders and drinkers. Birds were fed ad libitum a standard starter diet (22% crude protein (CP); 2900 kcal metabolizable energy (ME)/kg^−1^) from 0 to 4 weeks of age and then a grower diet (17% CP and 2850 kcal ME/kg^−1^) from 4 to 16 weeks of age. Quail were reared under a 24-h light photoperiod for 4 weeks, followed by a 14 h:10 h light:dark cycle.

### Trait records

Weekly body weight data of 805 quail (399 females and 406 males) from the parental, F_1_ and F_2_ generations were collected from hatching until 16 weeks of age (WK 0–16). A total number of 3990 eggs obtained from 399 females were used to assess egg-related traits. Egg-related traits were measured at two different egg production stages: at the beginning of the egg production stage (first stage) and at 12 weeks of age (second stage). The two stages are indicated with subscript letters ‘_1_’ and ‘_2_’ in the abbreviations of traits. The first five eggs from each female were evaluated at each developmental stage. External and internal egg-related traits, including egg weight (EW), egg long axis (ELA), egg short axis (ESA), eggshell strength (ESS), eggshell weight (ESW), egg equator thickness (EET), yolk weight (YW), yolk diameter (YD), yolk colour, lightness (L* value) (YC-L*), redness (a* value) (YC-a*), yellowness (b* value) (YC-b*), albumen weight (AW), age at first egg (AFE), total number of laid eggs from maturation up to 16 weeks of age (TLE), and egg production rate (EPR) were evaluated using the methodology described in our previous publication^17^. The average data from parental, F_1_ and F_2_ generations were subjected to one-way analysis of variance (ANOVA), followed by Tukey’s HSD test using JMP v. 11.0.2 (SAS Institute Inc., Tokyo, Japan).

### DNA collection and RAD library sequencing

Blood samples from all birds were collected using the method described by^56^, and genomic DNA was extracted from each collected sample using phenol–chloroform and DNeasy Blood & Tissue Kits (Qiagen, Venlo, Netherlands) according to the manufacturer's protocol. DNA was quantified using a Qubit 3.0 assay fluorometer (Thermo Fisher Scientific Inc. Waltham, MA, USA). DNA concentrations were adjusted to 20 ng/µL to ensure consistent measurements and were subsequently used for library preparation. Birds used for matings along with 505 F_2_ quail were used for RAD-seq. RAD libraries were prepared according to the method described by Ref.^57^. A 50-bp single-end adapter using *EcoRI* and *BglII* enzymes was performed on an Illumina HiSeq 2500 (Illumina, San Diego, CA, USA) for RAD library sequencing. RAD-seq read data were deposited in the DDBJ Sequence Read Archive (accession no. DRA011153) https://ddbj.nig.ac.jp/search. The RAD-seq reads were trimmed using the TrimGalore program (http://www.bioinformatics.babraham.ac.uk/projects/trim_galore/, accessed on 16 March 2020), and the trimmed reads were mapped onto the Japanese quail genome (GCA_001577835.1 *Coturnix japonica* 2.—NCBI) using Bowtie2 with default settings^58^. Further, the reads were converted to binary sequence alignment/map format (BAM) files using SAMtools^59^. Variant detection was initially performed for the F_1_ generation. The BAM files of F_1_ lines were processed using the SAMtools mpileup and varscan2 mpileup2cns^60^ with default parameters and then changed to min-coverage 5. The variant call format (vcf) files were merged with bcftools^61^, and the merged file was further screened using vcftools^62^ with the following parameters: minDP 5, min-meanDP 5, maxDP 100, min-alleles 2, max-alleles 2, and max-missing 1. The screened sites that were heterozygous for all F_1_ populations are summarised in the position list. Within the position list, polymorphisms of all samples, including birds from P_1_, P_2_, F_1_, and F_2_, were named using the SAMtools mpileup and varscan with the aforementioned parameters and merged using bcftools. After the polymorphism detection steps, only the GT fields were exported using vcftools and used for further analyses.

### Association analysis

Association analysis was performed using general linear model (GLM) and mixed linear model (MLM) approaches^63^ implemented by TASSEL v. 5^64^. GLM evaluation incorporated population structure (Principal component analysis, (PCA)) and MLM used both population structure (PCA) and kinship matrix in the association analysis. The PCA and covariance analysis were performed using the method reported by Ref.^65^ to describe the population structure. A kinship matrix was produced using the Kinship tool with the Scald_IBS method to avoid spurious associations due to relatedness and population structure. In the "Scald_IBS" method, genotypes were encoded based on the count of the alleles at that locus. By utilizing this encoded data, the kinship matrix was estimated. The efficient mixed-model association (EMMA) algorithm^66^ and population parameter previously determined (P3D) variance component estimation were implemented to decrease the computing time for a large dataset^67^. Statistically significant associations with *p*-values < P_threshold_ = 0.01/N, where N is the total number of SNP markers, were identified, and a standard correction was performed by applying a Bonferroni procedure at a minimum value of 3.88806E^−36^ for GLM and 7.03385E^−22^ for MLM^68^. The coefficient of determination (R^2^) was subsequently calculated to quantify the extent to which genetic markers explained the phenotypic variance. The significant value and the marker effect for each SNP were exported, and Manhattan as well as quantile–quantile (QQ) plots were generated in the R Project for Statistical Analysis v. 3.6.1^69^ using the qqman package^70^. The data were subjected to a correction procedure to account for the influence of fixed effects, specifically birth date, sex, and dame. This adjustment was performed using the least squares method in R-Project for Statistical Analysis v. 3.6.1^69^.

### Ethical approval

Animal care, experimental protocols, and blood sample collections were approved and conducted in accordance with the Rules on Experimental Animals and Animal Experiments at Hiroshima University, Graduate School of Integrated Sciences for Life, Laboratory of Animal Breeding and Genetics (Approval No. C20-15) and the protocol described in the Guidelines for Proper Conduct of Animal Experiments, Science Council, Japan https://www.scj.go.jp/ja/info/kohyo/pdf/kohyo-20-k16-2e.pdf. Also, we confirm that all methods have been conducted in adherence to the ARRIVE guidelines (https://arriveguidelines.org).

## Results

Filial generations obtained from reciprocal matings are presented separately. The body weights of female and male birds in the LS and NS strains and their F_1_ and F_2_ generations are shown in Tables 1 and 2, respectively. In females, LS F_1_ (LS♀ ✕ NS♂) quail showed the highest body weight at hatching (8.19 and 8.54 g, respectively). The lowest body weight was detected in NS quail from hatching to 16 weeks of age. No difference was found between F_2_ generations in WK 0–2. From 4 to 16 weeks of age, the LS strain presented the highest body weight compared to that of the other groups. In males, the LS F_1_ (LS♀ ✕ NS♂) quail showed the highest body weight at birth. However, no difference was observed between the weights of the other quail groups, except for NS. Moreover, no significant differences were observed between the F_1_ generations in WK 1–16. The NS quail showed the lowest body weight during the entire period analysed. The LS strain exhibited the highest body weight from 4 to 16 weeks of age compared to those observed at all generations.

**Table 1:** Body weight (mean ± standard error) of LS and NS female birds and their F_1_ and F_2_ hybrids from 0 to 16 weeks of age.

Weeks of age	LS (n = 50)	NS (n = 50)	F_1_ (LS♂ × NS♀) (n = 25)	F_1_ (LS♀ × NS♂) (n = 25)	F_2_ (LS♂ × NS♀) (n = 138)	F_2_ (LS♀ × NS♂) (n = 111)
WK 0	8.19 ± 0.11^a^	6.09 ± 0.07^d^	6.89 ± 0.19 ^c^	8.54 ± 0.12^a^	7.38 ± 0.07^b^	7.38 ± 0.05^b^
WK 1	27.21 ± 0.41^ab^	21.29 ± 0.36^d^	25.62 ± 0.49^bc^	28.52 ± 0.42^a^	24.20 ± 0.25^c^	24.02 ± 0.27^c^
WK 2	56.72 ± 0.95^a^	44.02 ± 0.58^d^	52.27 ± 1.07^b^	57.66 ± 0.71^a^	49.30 ± 0.50^bc^	47.47 ± 0.57^c^
WK 3	95.96 ± 1.45^a^	71.77 ± 0.72^e^	88.51 ± 1.61^b^	94.27 ± 0.99^ab^	80.83 ± 0.78^c^	77.36 ± 0.72^d^
WK 4	136.58 ± 1.66^a^	93.41 ± 0.94^e^	119.44 ± 1.41^b^	120.94 ± 1.39^b^	109.92 ± 0.89^c^	101.75 ± 0.92^d^
WK 5	156.48 ± 1.79^a^	110.25 ± 1.26^e^	140.06 ± 1.94^b^	144.68 ± 1.82^b^	126.73 ± 1.07^c^	122.57 ± 0.95^d^
WK 6	190.49 ± 2.18^a^	127.26 ± 1.69^e^	160.82 ± 2.40^b^	161.33 ± 2.40^b^	146.36 ± 1.20^c^	140.21 ± 1.21^d^
WK 7	206.44 ± 2.24^a^	133.40 ± 1.88^e^	165.45 ± 2.34^b^	164.41 ± 1.60^bc^	156.78 ± 1.27^c^	146.80 ± 1.28^d^
WK 8	211.60 ± 2.40^a^	133.69 ± 1.94^e^	165.55 ± 2.55^bc^	169.41 ± 2.02^b^	159.48 ± 1.25^c^	150.52 ± 1.27^d^
WK 9	214.86 ± 2.67^a^	138.86 ± 1.83^e^	166.14 ± 2.57^bc^	170.96 ± 1.83^b^	161.68 ± 1.27^c^	152.08 ± 1.29^d^
WK 10	219.96 ± 2.41^a^	141.96 ± 1.82^e^	173.47 ± 2.55^b^	175.55 ± 1.92^b^	164.16 ± 1.22^c^	155.42 ± 1.27^d^
WK 11	222.21 ± 2.69^a^	141.79 ± 1.70^e^	172.07 ± 2.49^bc^	178.06 ± 2.14^b^	165.54 ± 1.28^c^	155.83 ± 1.41^d^
WK 12	221.88 ± 2.73^a^	142.78 ± 1.93^d^	171.43 ± 2.85^b^	175.87 ± 2.45^b^	166.59 ± 1.25^b^	158.18 ± 1.38^c^
WK 13	222.30 ± 2.81^a^	142.90 ± 1.79^e^	173.98 ± 2.74^bc^	179.90 ± 2.51^b^	167.33 ± 1.25^c^	159.14 ± 1.42^d^
WK 14	224.78 ± 2.82^a^	145.01 ± 1.81^e^	173.89 ± 3.05^bc^	179.63 ± 2.46^b^	169.09 ± 1.29^c^	160.16 ± 1.45^d^
WK 15	226.11 ± 3.08^a^	143.52 ± 1.84^d^	173.80 ± 2.77^b^	178.86 ± 2.39^b^	169.64 ± 1.28^b^	161.41 ± 1.42^c^
WK 16	225.76 ± 3.13^a^	145.02 ± 1.89^e^	174.82 ± 2.84^bc^	178.51 ± 2.41^b^	168.15 ± 1.32^c^	161.61 ± 1.54^d^

^a–e^Means with different superscript letters are significantly different in each week of age (Tukey’s HSD test, *P* < 0.05).

WK 0–16, body weight at weeks of age.

The data of LS, NS, F_1_ (LS♂ ✕ NS♀), and F_1_ (LS♀✕NS♂) are the same as those in our previous paper^117^.

**Table 2:** Body weight (mean ± standard error) of LS and NS male birds and their F_1_ and F_2_ hybrids from 0 -16 weeks of age.

Weeks of age	LS (n = 50)	NS (n = 50)	F_1_ (LS♂ × NS♀) (n = 25)	F_1_ (LS♀ × NS♂) (n = 25)	F_2_ (LS♂ × NS♀) (n = 139)	F_2_ (LS♀ × NS♂) (n = 117)
WK 0	7.94 ± 0.11^a^	5.91 ± 0.10^c^	7.00 ± 0.21^b^	8.41 ± 0.14^a^	7.26 ± 0.08^b^	7.41 ± 0.05^b^
WK 1	24.93 ± 0.43^ab^	19.74 ± 0.44^d^	25.02 ± 0.50^abc^	26.70 ± 0.48^a^	23.34 ± 0.27^c^	23.53 ± 0.26^bc^
WK 2	51.52 ± 0.81^a^	40.58 ± 0.87^c^	52.32 ± 0.64^a^	53.79 ± 1.17^a^	46.70 ± 0.52^b^	46.64 ± 0.51^b^
WK 3	88.18 ± 1.28^a^	67.12 ± 1.02^c^	86.82 ± 1.08^a^	86.50 ± 1.45^a^	75.56 ± 0.75^b^	76.23 ± 0.78^b^
WK 4	126.25 ± 1.53^a^	86.71 ± 1.20^e^	112.91 ± 1.23^b^	111.81 ± 1.67^b^	102.63 ± 0.86^c^	99.03 ± 0.88^d^
WK 5	144.04 ± 1.72^a^	99.12 ± 1.13^d^	127.80 ± 1.41^b^	126.50 ± 2.13^b^	117.89 ± 0.96^c^	114.88 ± 0.93^c^
WK 6	161.08 ± 1.72^a^	105.18 ± 1.06^e^	139.47 ± 1.69^b^	135.30 ± 2.38^bc^	131.75 ± 0.97^c^	124.86 ± 1.00^d^
WK 7	172.50 ± 1.86^a^	108.53 ± 1.12^d^	143.10 ± 1.68^b^	138.07 ± 2.42^b^	136.40 ± 1.01^b^	129.76 ± 1.16^c^
WK 8	175.12 ± 1.80^a^	110.47 ± 1.19^d^	141.75 ± 1.99^b^	139.26 ± 2.32^b^	137.24 ± 1.04^b^	131.51 ± 1.27^c^
WK 9	177.95 ± 1.83^a^	112.97 ± 1.20^d^	143.71 ± 1.94^b^	141.55 ± 2.33^b^	138.53 ± 1.04^b^	133.27 ± 1.20^c^
WK 10	180.54 ± 1.98^a^	114.14 ± 1.29^d^	144.36 ± 2.01^b^	143.79 ± 2.45^b^	140.64 ± 1.03^b^	134.00 ± 1.25^c^
WK 11	183.77 ± 1.91^a^	115.66 ± 1.35^d^	146.21 ± 2.07^b^	144.66 ± 2.51^b^	141.57 ± 1.11^b^	136.08 ± 1.30^c^
WK 12	182.17 ± 1.84^a^	116.80 ± 1.35^d^	147.54 ± 2.08^b^	146.55 ± 2.56^b^	143.70 ± 1.07^b^	137.71 ± 1.21^c^
WK 13	183.26 ± 1.87^a^	117.57 ± 1.38^d^	148.61 ± 2.21^b^	146.98 ± 2.59^bc^	144.94 ± 1.08^b^	139.18 ± 1.26^c^
WK 14	185.29 ± 1.83^a^	120.02 ± 1.37^d^	149.14 ± 2.07^b^	147.31 ± 2.77^bc^	146.25 ± 1.05^b^	140.19 ± 1.21^c^
WK 15	186.94 ± 1.85^a^	120.38 ± 1.45^d^	150.30 ± 2.14^b^	148.31 ± 3.16^bc^	147.66 ± 1.08^b^	141.45 ± 1.23^c^
WK 16	188.01 ± 1.90^a^	121.13 ± 1.51^d^	150.31 ± 1.89^bc^	146.58 ± 3.19^bc^	149.12 ± 1.08^b^	142.69 ± 1.22^c^

^a–e^Means with different superscript letters are significantly different in each week of age (Tukey’s HSD test, *P* < 0.05).

WK 0–16, body weight at weeks of age.

The data of LS, NS, F_1_ (LS♂ ✕ NS♀), and F_1_ (LS♀ ✕ NS♂) are the same as those in our previous paper^117^.

Table 3 shows the means and standard errors for egg-related traits in the LS and NS strains of Japanese quail and their F_1_ and F_2_ hybrids in the first and second egg-laying stages. The LS strain EW, ESA, ESW, and AW exhibited the highest value among all groups in both egg-laying stages. NS females showed the highest values for the YC-a*_1_ and YC-b*_2_ traits across generations. F_2_ (LS♂ ✕ NS♀) females showed the lowest value in YC-a*_1_ (2.64) compared to the those observed in the other groups. No difference was observed in EET_1_ between the parental and filial generations. No significant differences were observed in ELA_1_ between NS and both filial generation females. Both F_2_ groups presented the lowest ESS_1,2_ among all groups, whereas no differences were observed between the parental and F_1_ quail in the first and second stage of egg production. F_2_ (LS♂ ✕ NS♀) birds started egg laying at a later age (49.01 days) than the other groups. F_1_ birds laid more eggs than the parental and F_2_ generations. Moreover, egg production in F_2_ was lower than that in the parental generation.

**Table 3:** Means and standard errors for egg-related traits in LS and NS strains of Japanese quail and their F_1_ and F_2_ hybrids.

Traits	LS (n = 50)	NS (n = 50)	F_1_ (LS♂ × NS♀) (n = 25)	F_1_ (LS♀ × NS♂) (n = 25)	F_2_ (LS♂ × NS♀) (n = 138)	F_2_ (LS♀ × NS♂) (n = 111)
Egg weight_1_ (g)	10.07 ± 0.11^a^	8.51 ± 0.11^bc^	9.00 ± 0.17^b^	9.06 ± 0.15^b^	8.62 ± 0.08^b^	8.22 ± 0.07^c^
Egg long axis_1_ (mm)	30.77 ± 0.14^a^	29.99 ± 0.18^b^	29.80 ± 0.21^b^	30.43 ± 0.20^ab^	29.75 ± 0.11^b^	29.98 ± 0.12^b^
Egg short axis_1_ (mm)	24.75 ± 0.09^a^	23.50 ± 0.10^bc^	23.75 ± 0.13^b^	23.15 ± 0.10^cd^	23.46 ± 0.07^bc^	22.83 ± 0.07 ^d^
Eggshell strength_1_ (kg/cm^2^)	1.39 ± 0.04^ab^	1.52 ± 0.04^a^	1.45 ± 0.03^ab^	1.35 ± 0.03^bc^	1.22 ± 0.02 ^d^	1.27 ± 0.02^cd^
Eggshell weight_1_ (g)	1.17 ± 0.01^a^	1.02 ± 0.01^bc^	1.08 ± 0.02^b^	1.09 ± 0.02^b^	0.98 ± 0.01^cd^	0.95 ± 0.01 ^d^
Egg equator thickness_1_ (mm)	0.28 ± 0.00^ab^	0.28 ± 0.00^b^	0.29 ± 0.00^a^	0.29 ± 0.00^ab^	0.29 ± 0.00^ab^	0.29 ± 0.00^a^
Yolk weight_1_(g)	3.08 ± 0.05^a^	2.90 ± 0.05^ab^	2.83 ± 0.05^bcd^	2.64 ± 0.06^cd^	2.84 ± 0.03^bc^	2.67 ± 0.03 ^d^
Yolk diameter_1_ (mm)	23.78 ± 0.15^ab^	23.13 ± 0.21^bc^	23.11 ± 0.19^bc^	22.55 ± 0.20^c^	23.98 ± 0.10^a^	23.18 ± 0.11^c^
Yolk colour-lightness_1_	56.23 ± 0.22^b^	56.51 ± 0.29^b^	58.32 ± 0.35^a^	59.25 ± 0.31^a^	55.78 ± 0.14^bc^	55.14 ± 0.17^c^
Yolk colour-redness_1_	9.07 ± 0.27^b^	10.53 ± 0.35^a^	5.33 ± 0.53^cd^	6.59 ± 0.41^c^	2.64 ± 0.23 ^e^	4.54 ± 0.23 ^d^
Yolk colour-yellowness_1_	36.52 ± 0.34^bc^	36.25 ± 0.36^c^	34.34 ± 0.37 ^d^	35.60 ± 0.40^cd^	38.25 ± 0.21^a^	37.57 ± 0.25^ab^
Albumen weight_1_ (g)	5.48 ± 0.07^a^	4.51 ± 0.06^c^	4.91 ± 0.09^b^	4.43 ± 0.07^c^	4.66 ± 0.04^bc^	4.53 ± 0.05^c^
Egg weight_2_ (g)	12.15 ± 0.12^a^	9.91 ± 0.12^cd^	10.85 ± 0.17^b^	11.07 ± 0.14^b^	10.06 ± 0.10^c^	9.54 ± 0.08 ^d^
Egg long axis_2_ (mm)	31.96 ± 0.14^ab^	31.44 ± 0.17^b^	31.66 ± 0.19^ab^	31.66 ± 0.21^ab^	32.04 ± 0.12^a^	32.03 ± 0.12^ab^
Egg short axis_2_ (mm)	26.18 ± 0.10^a^	24.09 ± 0.09 ^d^	25.18 ± 0.17^b^	25.12 ± 0.10^b^	24.57 ± 0.08^c^	23.89 ± 0.07 ^d^
Eggshell strength_2_ (kg/cm^2^)	1.48 ± 0.05^a^	1.52 ± 0.05^a^	1.48 ± 0.04^a^	1.45 ± 0.02^a^	1.21 ± 0.01^b^	1.28 ± 0.02^b^
Eggshell weight_2_ (g)	1.37 ± 0.02^a^	1.20 ± 0.02^bc^	1.26 ± 0.02^b^	1.27 ± 0.02^b^	1.15 ± 0.01^c^	1.10 ± 0.01 ^d^
Egg equator thickness_2_ (mm)	0.30 ± 0.00^ab^	0.28 ± 0.00^c^	0.31 ± 0.00^a^	0.30 ± 0.00^ab^	0.29 ± 0.00^bc^	0.29 ± 0.00^bc^
Yolk weight_2_(g)	3.77 ± 0.05^a^	3.26 ± 0.05^c^	3.53 ± 0.06^ab^	3.40 ± 0.06^bc^	3.70 ± 0.04^a^	3.35 ± 0.03^bc^
Yolk diameter_2_ (mm)	25.16 ± 0.13^b^	23.65 ± 0.15 ^d^	24.98 ± 0.16^bc^	24.27 ± 0.16^cd^	26.14 ± 0.11^a^	24.90 ± 0.10^bc^
Yolk colour-lightness_2_	58.46 ± 0.26^bc^	58.84 ± 0.29^b^	60.42 ± 0.35^a^	59.41 ± 0.26^ab^	58.60 ± 0.16^b^	57.77 ± 0.17^c^
Yolk colour-redness_2_	8.90 ± 0.26^a^	7.92 ± 0.37^ab^	6.24 ± 0.51^bc^	8.88 ± 0.38^a^	3.00 ± 0.26 ^d^	4.91 ± 0.20^c^
Yolk colour-yellowness_2_	41.08 ± 0.36^a^	41.23 ± 0.35^a^	38.22 ± 0.48^bc^	38.94 ± 0.34^b^	37.15 ± 0.22^cd^	36.37 ± 0.23 ^d^
Albumen weight_2_ (g)	6.27 ± 0.08^a^	5.13 ± 0.07^c^	5.71 ± 0.08^b^	5.32 ± 0.08^bc^	5.37 ± 0.06^bc^	5.24 ± 0.05^c^
Age at first egg	46.12 ± 0.51^b^	44.24 ± 0.92^bc^	42.28 ± 0.62^c^	40.68 ± 0.62^c^	49.01 ± 0.44^a^	46.56 ± 0.58^b^
Total laid eggs	56.90 ± 1.17^bc^	57.00 ± 1.43^bc^	59.24 ± 2.31^ab^	66.24 ± 0.99^a^	47.82 ± 0.78 ^d^	52.70 ± 1.15^c^
Egg production rate	0.86 ± 0.02^ab^	0.84 ± 0.02^b^	0.85 ± 0.03^ab^	0.93 ± 0.01^a^	0.76 ± 0.01^c^	0.80 ± 0.01^bc^

^a–e^Means with different superscript letters show the significantly different in all individuals (Tukey’s HSD test, *P* < 0.05).

_1,2_Subscript letters are first and second egg laying stages.

The data of LS, NS, F_1_ (LS♂ ✕ NS♀), and F_2_ (LS♂ ✕ NS♀) are the same as those in our previous paper^17^.

**Figure 1:**
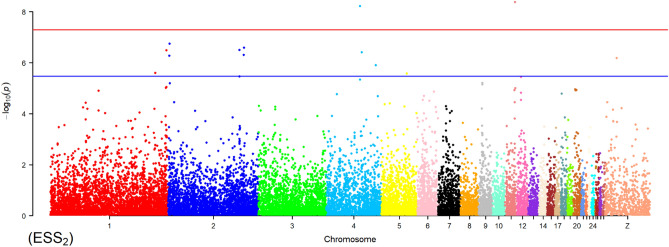
The Manhattan plot shows the association of SNP markers with ESS_2_ in the GLM. Each dot represents an SNP. The figure illustrates the level of statistical significance (y-axis) as measured by the negative log of the corresponding *p*-value for each SNP. Each SNPs type is indicated by dots of different colours, which are arranged by chromosomal location (x-axis). The horizontal red line indicates the threshold of 5% Bonferroni genome-wide significance (*p* = − log_10_ (5e−08)), and the underlined blue line presents a genome-wide suggestive at (*p* = − log_10_ (0.3e−05)).

**Figure 2:**
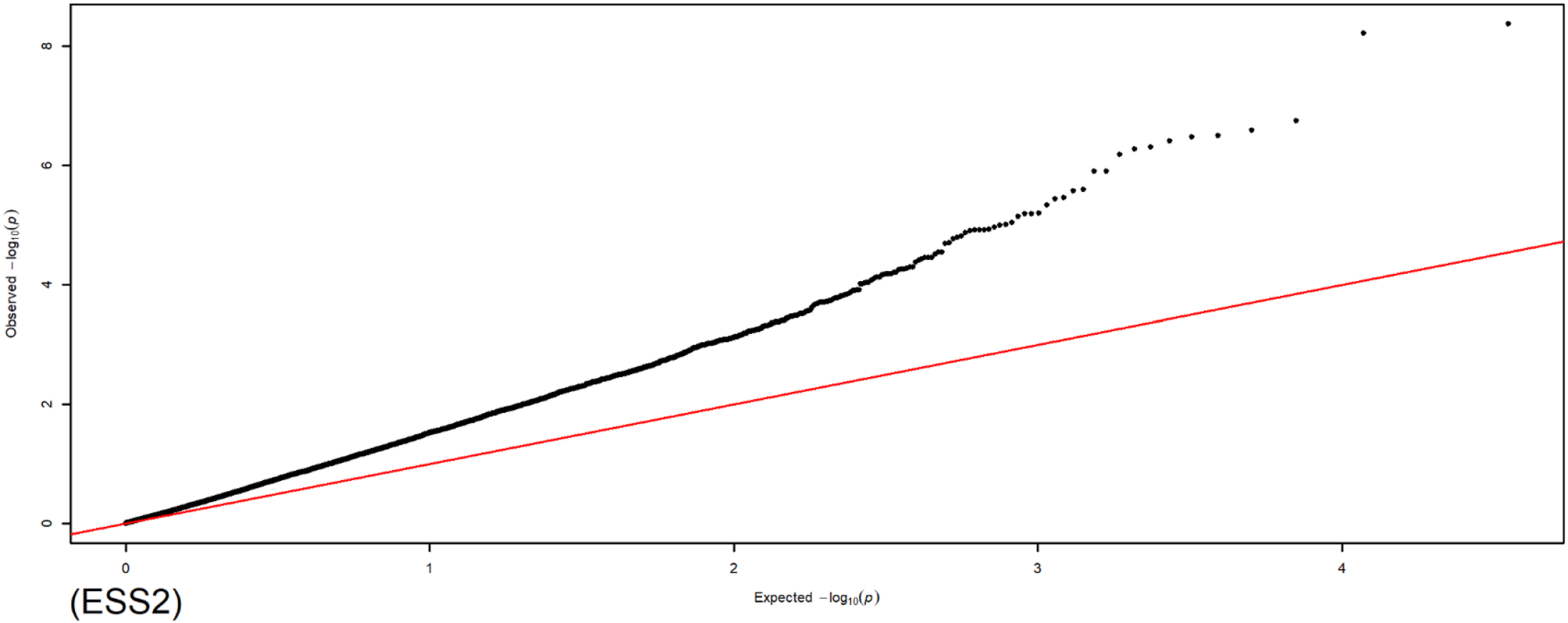
Quantile–quantile plot for ESS_2_ trait based on the GLM analysis.

Illumina HiSeq 2500 produced 733,233,057 RAD-seq reads for the treated samples. A total of 25,631 SNPs that passed the quality control were used for the GLM and MLM analyses. This subset of SNP genotypes was used to produce a kinship matrix for MLM analysis. The same subset of SNP markers was also used to perform the PCA in this study. General and mixed linear models produced 1,941 and 2,986 associations, respectively, after applying statistically significant loci with Bonferroni corrections. Statistically significant associations with *p*-values lower than the threshold were excluded, and 383 SNPs for 1,151 associations (Supplementary File S1) and 734 SNPs for 1,442 associations (Supplementary File S2) were identified to be significantly associated with production traits in the GLM and MLM approaches, respectively. GLM identified SNPs that were located on chromosomes 1–13, 15, 17–20, 24, 26–28, and Z, underlying all analysed traits except EW_1_, AW_1_, and YC-b*_2_ (Table 4). The maximum number of SNPs and associations was identified on the Z chromosome (122 and 611, respectively), followed by chromosome 1 (61 and 95, respectively) and chromosome 4 (52 and 92, respectively), while the minimum number of SNPs and associations (1 and 1, respectively) was found on chromosomes 13, 20, and 28 underlying WK 0, ESS_1_, and WK 3, respectively. A total of 1065 and 86 associations were detected for body weight and egg-related traits, respectively (Table 4). No associations for body weight traits were identified on chromosomes 12 (5,472,506 bp) and 20 (11,476,337 bp). Additionally, no associations for egg-related traits were detected on chromosomes 13, 24, and 26–28. The percentage of phenotypic variance explained by marker (R^2^) detected by the associations ranged between 0.003 and 0.097 using the GLM approach. Figures 1 and 2 represent the Manhattan and QQ plots for ESS_2_, respectively, wherein 14 associations were found on chromosomes 1, 2, 4, 5, 11, and Z. The Manhattan and QQ plots for the remaining traits from the GLM are shown in Supplementary File S4.

**Table 4:** Summary of chromosomes contained SNPs associations in production traits in GLM approach.

Chr. no.	SNP position/bp	Trait associated	Total SNPs	Association		Total associations
				Body weight	Egg traits	
1	957285–172994886	WK 0–16; EW_2_; ELA_1_; ESA_1,2_; ESS_2_; ESW_2_; EET_2_; YW_1,2_; YD_1,2_; YC-L*_2_; YC-a*_1,2_; AW_2_; AFE	61	70	25	95
2	1464677–131855473	WK 0–6, 9, 13–14, 16; ESS_1,2_; EET_2_; YC-L*_2_; YC-a*_1_; AFE; TLE	36	45	11	56
3	20855401–92027563	WK 0–16; EET_2_; YD_1,2_; AFE	24	50	5	55
4	3354007–73947532	WK 0, 2–5; ESS_2_; AFE; TLE; EPR	52	80	12	92
5	3353152–46814564	WK 0, 3, 5; ELA_2_; ESS_2_	8	7	2	9
6	8149405–21300272	WK 0, 3–5; YW_2_; TLE; EPR	10	16	3	19
7	10882063–25758848	WK 0, 2–16; TLE	12	42	1	43
8	1197173–24139329	WK 0, 2–4, 9–16; ESW_2_; EET_2_; YC-a*_1_	8	14	3	17
9	5538823–19764997	WK 0, 2–5; ESW_2_	11	22	2	24
10	2928963–16817587	WK 3, 5–6, 8–16; YC-L*_1_	3	12	1	13
11	9481215–14969883	WK 3; ESS_2_; YC-L*_2_	3	1	2	3
12	5472506–12775848	ESS_1_; ESW_1_	2	0	2	2
13	13271838	WK 0	1	1	0	1
15	8597788–11558238	WK 3, 6–16; ESW_1_; EET_1_	10	69	2	71
17	1771577–6668466	WK 0, 3–5; YC-L*_1_	5	7	1	8
18	5637999–8429549	WK 3; ELA_2_; EET_1_	4	2	2	4
19	3503431–4877714	WK 5; YC-L*_2_	2	1	1	2
20	11476337	ESS_1_	1	0	1	1
24	3735689	WK 2, 3	1	2	0	2
26	270586–287070	WK 8-16	2	18	0	18
27	1662182–1981112	WK 0, 2–4	4	4	0	4
28	337525	WK 3	1	1	0	1
Z	1871013–66979967	WK 0, 1, 4–16; ESS_1,2_; ESW_1_; YC-L*_1_; YC-b*_1_; AFE; TLE; EPR	122	601	10	611

WK 0–16, body weight at weeks of age; _1,2_subscript letters mean the first and second egg laying stages.

*EW* egg weight, *ELA* egg long axis, *ESA* egg short axis, *ESS* eggshell strength, *ESW* eggshell weight, *EET* egg equator thickness, *YW* yolk weight, *YD* yolk diameter, YC-L* yolk colour-lightness, YC-a* yolk colour-redness, YC-b* yolk-colour-yellowness, *AW* albumen weight, *AFE* age at first egg, *TLE* total laid eggs, *EPR* egg production rate.

**Figure 3:**
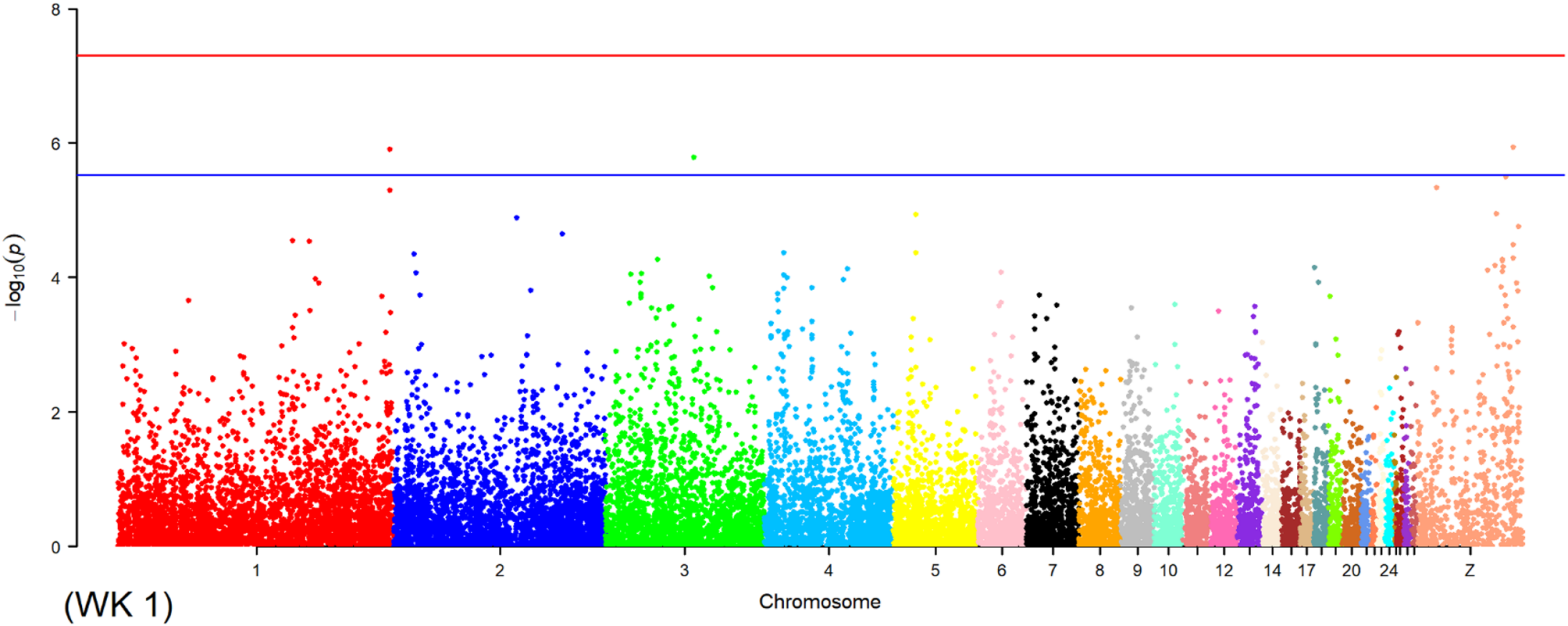
The Manhattan plot shows the association of SNP markers with body weight at WK 1 in the MLM. Each dot represents an SNP. The figure illustrates the level of statistical significance (y-axis) as measured by the negative log of the corresponding *p*-value for each SNP. Each SNPs type is indicated by dots of different colours, which are arranged by chromosomal location (x-axis). The horizontal red line indicates the threshold of 5% Bonferroni genome-wide significance (*p* = − log_10_ (5e−08)), and the underlined blue line presents a genome-wide suggestive at (*p* = − log_10_ (0.3e−05)).

**Figure 4:**
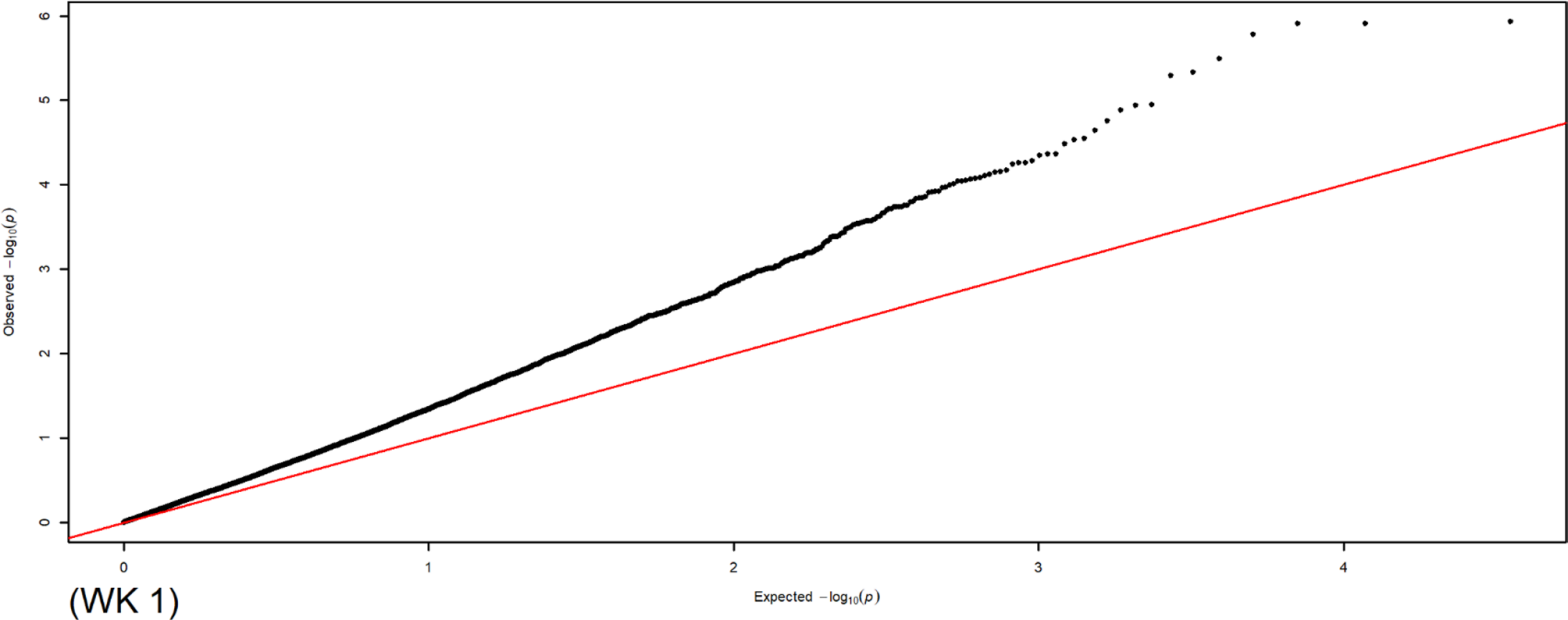
Quantile–quantile plot for body weight at WK 1 based on the MLM.

As shown in Table 5, MLM recognised SNPs that were positioned on all chromosomes except for chromosome 16 and associated with all traits except for ELA_2_. The maximum number of associations was detected on the Z chromosome (N = 516), followed by chromosomes 1 (N = 208), 3 (N = 105), and 2 (N = 96), while the minimum number of associations was observed on chromosome 25 (N = 1) for YC-L*_2_ and chromosome 28 (N = 1) for egg production rate. However, a maximum number of 149, 97, 76, and 69 SNPs were distributed on chromosomes 1, Z, 2, and 3, respectively. Following MLM and Kinship analyses, 739 and 703 associations were observed for body weight and egg-related traits, respectively. No SNP markers on chromosomes 11–12, 19–23, 25, and 28 were associated with body weight. The percentage of phenotypic variance detected by the associations ranged between 0.0004 and 0.1401 in the MLM analysis. The Manhattan and QQ plots for WK 1 are shown in Figs. 3 and 4, respectively. For WK 1 trait, four associations were detected on chromosomes 1 (N = 2), 3 (N = 1), and Z (N = 1). MLM plots for each Manhattan and QQ (Supplementary File S5) were plotted for the associated body weight and egg-related traits.

**Table 5:** Summary of chromosomes contained SNPs associations in production traits in MLM approach.

Chr. no.	SNP position/bp	Trait associated	Total SNPs	Association		Total associations
				Body weight	Egg traits	
1	9916560–174101702	WK 0–1, 3–16; EW_1,2_; ELA_1_; ESA_2_; ESS_1,2_; ESW_1,2_; EET_1,2_; YW_1,2_; YD_1,2_; YC-L*_1,2_; YC-a*_1,2_; YC-b*_1,2_; AW_1,2_; AFE; TLE; EPR	149	43	165	208
2	1888696–131855473	WK 2–5, 8–9; EW_1,2_; ELA_1_; ESA_1_; ESS_1,2_; ESW_1_; EET_1,2_; YW_1,2_; YD_1,2_; YC-L*_2_; YC-a*_1,2_; YC-b*_1_; AW_1,2_; AFE; TLE; EPR	76	25	71	96
3	1048779–100834547	WK 0–1, 4–16; EW_1_; ELA_1_; ESA_1,2_; ESS_1_; ESW_1,2_; EET_2_; YW_1,2_; YD_1,2_; YC-L*_1,2_; YC-a*_1,2_; YC-b*_1,2_; AW_1_; AFE; TLE; EPR	69	25	80	105
4	3252344–81870624	WK 2–5; EW_1_; ESS_1,2_; ESW_1_; EET_1_; YW_1_; YD_1_; YC-L*_1,2_; YC-a*_1,2_; YC-b*_1,2_; TLE; EPR	59	25	53	78
5	1071356–51998013	WK 4–5; EW_1_; ELA_1_; ESA_1_; ESS_2_; EET_2_; YW_1,2_; YC-L*_1_; YC-a*_1,2_; YC-b*_1,2_; AFE; TLE	36	3	41	44
6	2079850–27664331	WK 2–5; ELA_1_; ESS_1_; EET_2_; YW_1,2_; YD_1_; YC-L*_1,2_; YC-a*_1,2_; YC-b*_2_; EPR	20	11	20	31
7	227226–32854252	WK 0, 3, 6, 8–11, 13–15; EW_1_; ESA_1_; ESS_1,2_; ESW_2_; EET_1,2_; YW_2_; YD_1,2_; YC-L*_1,2_; YC-a*_1,2_; YC-b*_1,2_; EPR	34	19	34	53
8	1197173–26398286	WK 2, 4; ESS_1_; EET_2_; YD_1_; YC-L*_2_; YC-a*_2_; YC-b*_1,2_; AW_1_; AFE	17	2	15	17
9	3655608–19764997	WK 0, 2–4; EW_1,2_; ESA_1,2_; ESS_1,2_; ESW_2_; EET_2_; YW_1,2_; YC-L*_2_; YC-a*_1_; AW_1_; TLE; EPR	25	11	27	38
10	2928963–16720330	WK 10, 13–16; EET_1_; YW_1_; YD_1_; YC-L*_2_; YC-a*_1,2_; YC-b*_1_; TLE; EPR	19	5	22	27
11	2225815–14703022	EW_2_; ELA_1_; EET_1,2_; YW_2_; YC-L*_1,2_; YC-b*_1_; TLE; EPR	13	0	15	15
12	1170128–15591439	EW_1_; ESS_1,2_; EET_1,2_; YW_2_; YC-a*_2_; YC-b*_1,2_; AFE; EPR	20	0	21	21
13	4065467–13271838	WK 0; EET_1_; YW_1_	4	1	3	4
14	2318659–8360575	ELA_1_; ESW_1_; EET_1,2_; YC-a*_1,2_; AW_1_; EPR	12	0	13	13
15	2445568–11558238	WK 3, 6–16; ELA_1_; ESS_2_; EET_2_; YD_1,2_; YC-L*_2_; YC-a*_2_; YC-b*_1_	18	65	13	78
17	851671–7964836	WK 3–5; YC-L*_2_; YC-a*_2_; YC-b*_1,2_; TLE	11	3	8	11
18	825795–8429549	WK 2–3, 5; ESA_1_; YC-a*_2_	5	3	4	7
19	2778245–7475932	EET_1_; YW_2_; YC-L*_1_; YC-a*_2_; AFE	10	0	11	11
20	10082837–11107159	YW_2_; YD_2_; YC-b*_2_	3	0	5	5
21	1178424–4470040	EW_1_; ELA_1_; ESS_1_; YC-L*_2_; YC-a*_2_; YC-b*_2_; AW_1_; AFE; TLE	10	0	16	16
22	752464–1520677	TLE; EPR	5	0	7	7
23	2922410–4968083	EW_1_; ELA_1_; ESA_1_; YC-L*_2_; AW_1_	4	0	7	7
24	2420764–3735689	WK 2–3; EPR	2	2	1	3
25	1683907	YC-L*_2_	1	0	1	1
26	270586–3873130	WK 8–16; EW_1_; ESA_1_; ESW_1_; YW_1_; YD_1_; YC-b*_2_; TLE; EPR	8	10	10	20
27	1598345–2829862	WK 0; YD_2_; YC-a*_2_; TLE; EPR	6	1	8	9
28	2708993	EPR	1	0	1	1
Z	625881–66979967	WK 1, 4, 6–16; EW_1,2_; ELA_1_; ESA_1_; EET_1_; YW_1,2_; YD_1_; YC-a*_2_; AW_1_; TLE	97	485	31	516

Trait abbreviations are presented in Table 4.

The combination of association markers detected in both the GLM and MLM analyses is explained in Table 6. A total of 191 SNPs associated with body weight and egg-related traits were shared in both models. Markers associated with all body weight traits were identified in the GLM and MLM analyses. However, no markers were found to be associated with EW_1_, ELA_2_, ESW_1_, EET_1_, YC-b*_2_, AW_2_ in both models, and EW_2_, ESA_1,2_, YD_2_, YC-L*_1_, YC-a*_1,2_, AW_1_, TLE, and EPR based on the GLM. In the GLM, 18 SNP markers were identified on chromosome 1 for ELA_1_ (N = 1), ESW_2_ (N = 4), EET_2_ (N = 1), YW_1,2_ (N = 1,1), YD_1_ (N = 1); chromosome 2 for ESS_2_ (N = 2); chromosome 4 for AFE (N = 1); chromosome 5 for ESS_2_ (N = 1); chromosome 9 for ESW_2_ (N = 2); chromosome 12 for ESS_1_ (N = 1); chromosome 19 for YC-L*_2_ (N = 1); and the Z chromosome for YC-L*_1_ (N = 1). Alternatively, in the MLM, 45 SNPs were observed on chromosome 1 for EW_2_ (N = 3), ESW_2_ (N = 5), and YW_1,2_ (N = 1,1); chromosome 2 for ESS_2_ (N = 2) and TLE (N = 1); chromosome 3 for TLE (N = 3) and EPR (N = 3); chromosome 5 for ELA_1_ (N = 1) and ESS_2_ (N = 1); chromosome 7 for YC-L*_1_ (N = 2); chromosome 8 for AW_1_ (N = 1); chromosome 9 for EW_2_ (N = 2), ESA_2_ (N = 1), ESW_2_ (N = 1), EET_2_ (N = 2), YW_2_ (N = 1), and YC-a*_1_ (N = 1); chromosome 12 for ESA_1_ (N = 1); chromosome 15 for YC-b*_1_ (N = 1); chromosome 19 for AFE (N = 1); chromosome 27 for TLE (N = 1) and EPR (N = 1); and Z chromosome for ELA_1_ (N = 3), ESA_1_ (N = 1), YC-a*_2_ (N = 1), and TLE (N = 3). Among the detected SNPs, 66 markers were associated with the same traits in both models across all chromosomes.

**Table 6:** SNP markers found in both GLM and MLM approaches.

No.	Position (bp)	Chr.	GLM		MLM		No.	Position (bp)	Chr.	GLM		MLM	
			Trait	*P*	Trait	*P*				Trait	*P*	Trait	*P*
1	9916560	1	WK 2–3	**	WK 3	*	97	19764997	9	WK 4–5	**	EET_2_	**
2	9994051	1	YW_2_	**	EW_2_; ESW_2_	**	98	2928963	10	WK 8–16	**	WK 10, 13–16	**
3	11027778	1	ESW_2_	**	EW_2_; ESW_2_	**	99	5472506	12	ESS_1_	**	ESS_1_	*
4	11027787	1	ESW_2_	**	EW_2_; ESW_2_	**	100	13271838	13	WK 0	**	WK 0	**
5	11033333	1	ESW_2_	*	ESW_2_	**	101	8597788	15	WK 3	**	WK 3	**
6	21594364	1	WK 5; EET2	**	WK 4	*	102	11549443	15	WK 8–16	**	WK 8–11, 13–16	**
7	33878341	1	WK 3	**	WK 3	**	103	11555370	15	WK 7–16	**	WK 7–16	**
8	53980437	1	WK 3	**	WK 3	**	104	11555372	15	WK 7–16	**	WK 7–16	**
9	105167226	1	WK 4	**	WK 4	**	105	11555374	15	WK 7–16	**	WK 7–16	**
10	125878827	1	ELA_1_; YW_1_; YD_1_	**	YW_1_; YD_1_	**	106	11555409	15	WK 6–16	**	WK 6–16; YC-b*_1_	**
11	129225772	1	WK 4	**	WK 4	*	107	11555410	15	WK 6–16	**	WK 6–16; YD_1_	**
12	129225785	1	WK 4	**	WK 4	*	108	11558238	15	WK 10–16	**	WK 13–16	**
13	147490325	1	WK 4	**	WK 4	*	109	1771577	17	WK 3, 5	**	WK 3	**
14	147607172	1	WK 0	**	WK 0	*	110	1819780	17	WK 0, 4–5	**	WK 4	**
15	150370977	1	WK 4–5	**	WK 4–5	**	111	5282689	17	WK 5	**	WK 5	**
16	150796680	1	ESW_2_	**	ESW_2_	*	112	8429549	18	WK 3	**	WK 2–3, 5	**
17	150937933	1	WK 3–5	**	WK 4	**	113	3503431	19	YC-L*_2_	*	AFE	**
18	152602081	1	WK 4–16	**	WK 4–16	**	114	3735689	24	WK 2–3	**	WK 2–3	**
19	153044456	1	WK 5–8, 13–14, 16	**	WK 5–8, 13–16	**	115	270586	26	WK 8–16	**	WK 8–16	**
20	158128424	1	WK 3	**	WK 3	**	116	287070	26	WK 8–16	**	WK 8	**
21	167714787	1	WK 0, 2, 5	**	WK 0	**	117	1662202	27	WK 2	**	TLE; EPR	**
22	170781102	1	WK 2, 4–5	**	WK 4–5	**	118	1981112	27	WK 0	**	WK 0; YD_2_	*
23	170781104	1	WK 4–5	**	WK 4–5	**	119	4231250	Z	WK 10, 14	**	WK 14	**
24	172994886	1	WK 1	*	YW_2_	**	120	5068401	Z	WK 10	**	WK 10; TLE	**
25	1888697	2	ESS_2_	**	ESS_2_	**	121	5068437	Z	WK 10	**	WK 10; TLE	**
26	7036370	2	WK 3–5	**	WK 3–4	**	122	5154957	Z	WK 10	**	WK 10; TLE	**
27	8090642	2	WK 3–5	**	WK 3–4	**	123	14969758	Z	WK 12	**	WK 12	**
28	8328071	2	WK 4	**	WK 4	*	124	15141498	Z	WK 7–14, 16	**	WK 7–8, 10–12	**
29	14123076	2	WK 3–4	**	WK 4	*	125	15453956	Z	WK 9–12, 16	**	WK 10	**
30	16656930	2	WK 2–3	**	WK 2–3	**	126	17867349	Z	WK 9–16	**	WK 12	**
31	19908441	2	WK 4	**	WK 4–5	*	127	27931470	Z	WK 6–13	**	WK 6–13	**
32	27448173	2	WK 4–6	**	WK 4	**	128	28077814	Z	WK 6–16	**	WK 6–16	**
33	77567411	2	WK 3	**	WK 3	**	129	28148932	Z	WK 7–12	**	WK 10	**
34	78192508	2	WK 0–3	**	WK 2–3	**	130	28308035	Z	WK 8–12	**	WK 7–8, 10–13	**
35	80286556	2	WK 5	**	WK 5	**	131	28900115	Z	WK 6–16	**	WK 6–16	**
36	84686112	2	WK 3–5	**	WK 4–5	**	132	29382006	Z	WK 12	**	WK 12	**
37	99126598	2	WK 3–5	**	WK 3, 5	**	133	29382008	Z	WK 12	**	WK 12	**
38	106857032	2	WK 0, 2–3	**	WK 2–3	**	134	29485342	Z	WK 6–16	**	WK 6–16	**
39	112648472	2	ESS_2_	**	ESS_2_; TLE	**	135	29600028	Z	WK 6–16	**	WK 6–16	**
40	131855473	2	WK 9, 14	**	WK 8, 9	**	136	29718460	Z	WK 9–16	**	WK 7–16	**
41	20855401	3	WK 5	**	WK 5	**	137	29718467	Z	WK 6–16	**	WK 6–16	**
42	20855430	3	WK 5	**	WK 5	**	138	30238353	Z	WK 9–16	**	WK 7, 9–16	**
43	22362768	3	WK 1, 3–5	**	WK 5	**	139	30848955	Z	WK 6–16	**	WK 6–16	**
44	22362804	3	WK 1, 3–5	**	WK 4–5	**	140	31163019	Z	WK 6–16	**	WK 6–16	**
45	22362805	3	WK 1, 3–5	**	WK 4–5	**	141	31203382	Z	WK 6–8, 10–13	**	WK 6–13	**
46	26848584	3	WK 2–3, 5	**	WK 5	**	142	31279475	Z	WK 7–16	**	WK 7–8, 10, 12–16	**
47	29553506	3	WK 0, 5	**	WK 0	**	143	31853927	Z	WK 6–16	**	WK 6–16	**
48	40472890	3	WK 0	**	WK 0	**	144	32023812	Z	WK 6–8, 11–12	**	WK 6–8, 10–13	**
49	44367043	3	WK 14–15	**	TLE; EPR	**	145	32143766	Z	WK 6–13, 15	**	WK 6, 8, 11–13	**
50	44367047	3	WK 14	**	TLE; EPR	**	146	32143773	Z	WK 7–16	**	WK 7–13	**
51	44367051	3	WK 9–10, 12–16	**	TLE; EPR	**	147	32656468	Z	WK 6–16	**	WK 6–16	**
52	44367054	3	WK 6–16	**	WK 6–16	**	148	32886446	Z	WK 7–16	**	WK 6–16	**
53	56080001	3	WK 1	**	WK 1	*	149	32899766	Z	WK 6–16	**	WK 6–16	**
54	3354007	4	WK 4	**	WK 4	**	150	33316240	Z	WK 6–13	**	WK 6–13	**
55	12472037	4	WK 2–3, 5	**	WK 2–3	**	151	33419905	Z	WK 6–16	**	WK 6–16	**
56	29632136	4	WK 2–5; AFE	**	WK 3–5	**	152	33878609	Z	WK 5–16	**	WK 6–16	**
57	30310512	4	WK 2–5	**	WK 3–4	**	153	36774490	Z	WK 7–16	**	WK 8–16	**
58	30310521	4	WK 3–4	**	WK 3	*	154	37786779	Z	WK 6–16	**	WK 6–16	**
59	30374577	4	WK 3,5	**	WK 3	*	155	39252909	Z	WK 7–16	**	WK 7–16	**
60	30374590	4	WK 3,5	**	WK 3	*	156	40386793	Z	WK 8–12, 14–16	**	WK 10	**
61	30468252	4	WK 3–5	**	WK 5	**	157	43128580	Z	WK 12	**	WK 10–13	**
62	30656142	4	WK 3–5	**	WK 3–5	**	158	43571244	Z	WK 11–16	**	WK 8, 12–14, 16; YD_1_	**
63	31129359	4	WK 3–5	**	WK 3–4	*	159	43581088	Z	WK 10–16	**	WK 8, 12–14, 16	**
64	50742079	4	WK 2–3, 5	**	WK 3	*	160	44810990	Z	WK 8–10, 12–16	**	WK 8, 10, 14	**
65	51668123	4	WK 3	**	WK 3	*	161	45427482	Z	WK 11–12	**	WK 8, 10–14	**
66	53250099	4	WK 2–3, 5	**	WK 3	**	162	45911151	Z	WK 11–12, 14–16	**	WK 7–8, 10–16	**
67	53319229	4	WK 2–3, 5	**	WK 3	**	163	47845702	Z	WK 11–12	**	WK 6–7, 10–13; YC-a*_2_	**
68	53780465	4	WK 3–5	**	WK 3, 5	**	164	48348063	Z	WK 7–16	**	WK 8–9, 13–16	**
69	53780471	4	WK 3, 5	**	WK 3	**	165	49154399	Z	WK 7–12	**	WK 6–13	**
70	55146141	4	WK 3, 5	**	WK 3	*	166	49154401	Z	WK 7–12	**	WK 6–13	**
71	14763102	5	WK 5	**	WK 4–5	**	167	54124844	Z	WK 9, 11–16	**	WK 12–16	**
72	19651280	5	WK 0	**	ELA_1_	**	168	54174428	Z	WK 13–16	**	WK 14–16	**
73	38217136	5	ESS_2_	*	ESS_2_	**	169	54174431	Z	WK 13–16	**	WK 14–16	**
74	44054239	5	WK 5	**	WK 5	**	170	54693463	Z	WK 10–13	**	WK 8, 10–13, 15	**
75	9709930	6	WK 0, 4–5	**	WK 4	*	171	55338268	Z	WK 9–16	**	WK 7–8, 10, 12, 14–15	**
76	10481155	6	WK 3–5	**	WK 3, 5	**	172	57426680	Z	WK 8–16	**	WK 7–16	**
77	10481196	6	WK 3–5	**	WK 3–5	**	173	57426684	Z	WK 7–16	**	WK 7–16	**
78	10680098	6	WK 5	**	WK 5	**	174	57470300	Z	WK 10–13, 15; YC-b*_1_	**	WK 10, 12	**
79	21300272	6	WK 3–5	**	WK 2–5	**	175	57635698	Z	WK 7–16	**	WK 7–16	**
80	10882063	7	WK 3	**	YC-L*_1_	**	176	57818905	Z	WK 8–16	**	WK 8–10, 12–16	**
81	18217205	7	WK 3–5	**	WK 3	**	177	57953545	Z	WK 0, 7–16	**	WK 7–16	**
82	21130444	7	WK 0	**	YC-L*_1_	**	178	58471010	Z	WK 10, 13	**	WK 10, 13	**
83	22657165	7	WK 6, 8–16	**	WK 6, 8–11, 13	**	179	58471020	Z	WK 7–16	**	WK 7–16	**
84	22657168	7	WK 6–16	**	WK 6, 8, 10–11, 13–15	**	180	58904207	Z	WK 13–16	**	WK 15–16	**
85	22657172	7	WK 8–16	**	WK 6, 8–9, 11	**	181	59494323	Z	WK 8–16	**	WK 7–16	**
86	1197173	8	WK 2–3	**	WK 2	*	182	61039048	Z	WK 1, 4, 7–16	**	WK 1, 4, 6–16; ESA_1_	**
87	4683580	8	WK 4	**	WK 4	**	183	61509499	Z	WK 7–16	**	WK 7–16	**
88	5974879	8	WK 0	**	AW_1_	**	184	61892165	Z	WK 9–15	**	WK 9–14	**
89	5538823	9	ESW_2_	**	EW_2_; ESA_2_; YW_2_	**	185	62313966	Z	WK 4, 7–16	**	WK 4, 6–16	**
90	5610382	9	ESW_2_	**	EW_2_; ESW_2_; YC-a*_1_	**	186	62604224	Z	WK 13	**	WK 14	**
91	6609081	9	WK 2–4	**	WK 2–4	**	187	63729978	Z	WK 11–12	**	WK 11–12	**
92	10286581	9	WK 2–4	**	WK 3–4	**	188	64124896	Z	WK 12	**	WK 12	**
93	10286589	9	WK 2–4	**	WK 3–4	**	189	66433809	Z	WK 9–16	**	WK 10–12; ELA_1_	**
94	10484278	9	WK 2–5	**	WK 3–4	**	190	66979966	Z	WK 10–12	**	WK 10–12; ELA_1_	**
95	16397647	9	WK 4–5	**	WK 4	**	191	66979967	Z	WK 10–12	**	WK 10–12; ELA_1_	**
96	19764973	9	WK 4-5	**	EET_2_	**							

Trait abbreviations are presented in Table 4; significant *P* values are indicated at level of **1% and *5%.

## Discussion

The evaluation of 44 phenotypic traits from 567 birds in the F_2_ revealed 383 SNPs with 1151 associations and 734 SNPs with 1442 associations for GLM and MLM approaches, respectively. Our identified SNPs were associated with all targeted traits except EW_1_, AW_1_, and YC-b*_2_ in the GLM and ELA_2_ in the MLM analysis. To the best of our knowledge, no such association analysis of production traits in Japanese quail have been reported using GLM and MLM approaches with RAD-seq data. However, several studies on QTL analysis of production traits in Japanese quail have been reported. Reference^20^ recognised QTLs underlying body weight at hatching and at four weeks of age on chromosome 1 at 12–13 cM. Here, we identified associations on the same chromosome at 11.9 cM for GLM and 12.38 cM for MLM corresponding for WK 0 and TLE traits, respectively. Reference^21^ reported QTL associated with body weight from hatching to three weeks of age on chromosome 3 between 27 and 30 cM. The results of the present study revealed trait associations for SNPs located between 28.73 and 37.74 cM on chromosome 3 in both GLM and MLM analyses. Moreover, using GLM and MLM approaches, we showed that associations underlying body weight traits were mostly located on chromosomes 1–10 and Z. Associations affecting hatching weight were identified on chromosomes 1–9, 13, 17, 27, and Z for GLM, and 1, 3, 7, 9, 13, and 27 for MLM approaches. Detected SNPs located at 20.16 cM on chromosome 7 were associated with hatching weight in both GLM and MLM analyses, and these results were consistent with the previously identified location of QTLs associated with this trait on chromosome 5 (19–20 cM)^22^. The detected QTL affecting liveweight measurement at week 5 has been shown to be located on chromosome 1 in a study investigating the genetic mapping of QTLs affecting body weight^18^, which is in agreement with the SNPs associated with body weight and located on chromosome 1 in our GLM and MLM analyses. Reference^3^ identified a QTL for body weight at 4 weeks of age at the centromere of chromosome 2 which is consistent with the locus detected for this trait on chromosome 2 in both association analyses performed in the present study. The study detected three QTLs associated with body weight at 1, 4, and 6 weeks of age, located in the initial region of chromosome 2 between 0 and 15 cM. Reference^2^ reported putative pleiotropic loci on chromosome 3 (52.6–56.7 Mb) affecting both weight and egg traits, where a QTL for egg weight co-localised with a QTL for body weight at 65 days of age. Interestingly, our results indicated an association in the same chromosome (56.08 Mb) controlling WK 1 in both the GLM and MLM analyses. This candidate region could underlie the genetic correlation already observed in quail between body weight and egg traits^71–73^. QTLs for early and late growth stages (5 and 70 weeks of age) have been previously detected on chromosome 1, positioned 18–19 cM^24^. In our MLM analysis, YW associations were identified on chromosome 1 between 17.68 and 21.16 cM. It is likely that the QTLs for BW and YW located on chromosome 1 represent a single gene. In addition to QTLs for body weight traits, Ref.^24^ identified QTLs for eggshell weight on chromosomes 1 (191 cM), 5 (12 cM), and 20 (21 cM); egg weight, egg number until the age of 69 weeks, and age of first egg on chromosome 6 positioned at 0, 32, and 34 cM, respectively. The results of the present study revealed associations for YC-L*_2_ and YC-a*_2_ on chromosome 1 (190.34 cM) as well as TLE and EPR on chromosome 6 (31.8 cM) in GLM. Alternatively, we identified EET_2_ QTLs on chromosome 5 (12.02 cM), YW_2_ on chromosome 6 (0 cM), and YW_2_ as well as YD_2_ on chromosome 20 (20.35 cM) in the MLM. These findings are consistent with the results presented by Ref.^24^. In turn, Ref.^3^ recognised QTLs for the number of eggs laid and the egg production rate on chromosome 1 between 36 and 42 cM. The differences among the results of these studies may be explained by the different durations of egg production. In our previous report, we identified a QTL for growth-related traits on chromosome 1 using the same Japanese quail strains^16^. Similarly, in the present study, two associations were found on the same locus (150,370,977 bp) on chromosome 1 for BW 4 and 5. In addition, we detected QTLs for ESA_1_ and YD_1_ on the Z chromosome between 22,757,726 and 31,279,475 bp^17^. Associations underlying WK 9 in GLM, EET_1_ in MLM, and WK 7–16 in both approaches were identified within the same chromosome and at the same positions. This was expected as positive correlations have been observed between the related egg trait and body weight^73^. Only chromosomal regions were explored in the above-mentioned studies on the Japanese quails; therefore, future studies focusing on the association between SNPs and candidate genes would be of great value for improving QTL resolution in Galliformes.

GWASs are widely used to distinguish SNPs associated with production traits in chickens^74,75^. In the first study of its kind on Japanese quail, we used association analysis with GLM and MLM, based on RAD-seq SNPs, to examine production traits like body weight and egg-related traits. Comparative genomic studies of Japanese quail and chickens based on cytogenetics^53^, orthologous genes^76^, and linkage analysis^52^ have shown a high rate of synteny-conserved karyotypes and genomic structure between these species. Therefore, the GWAS results from chickens were used to identify potential candidate genes in the present study. However, due to differences in growth conditions between chickens and quail, body weight at the early growth stage, sexual maturity age, and maximum growth rate were considered in both species.

The detection of associations related to body weight during juvenile age is likely due to the strong correlation between body weight traits during early growth stages. It is anticipated that numerous genes control body weight during this period because growth is a highly intricate trait influenced by multiple loci that affect appetite, feed intake, body composition, nutrient utilization, and physical activity. In the present study, six shared SNPs between GLM and MLM approaches were identified on chromosomes 1 (147.6 and 167.7 Mb), 3 (29.5 and 40.4 Mb), 13 (13.2 Mb), and 27 (1.9 Mb) associated with body weight at hatching, a trait that is strongly influenced by maternal effects^71^. Reference^77^ identified a chicken QTL on chromosome 27 in the same region as the one discovered in the present study. This region on chromosome 27 contains two genes, LIM and SH3 protein 1 (*LASP1*) and phosphatidylinositol-5-phosphate 4-kinase, type II, beta (*PIP4K2B*), which regulate the decrease in mice body weight^78,79^. Associations between gene expression at the initial ages have also been reported in chickens. Reference^80^ identified a genomic region (169.8–175.3 Mb) on chromosome 1 associated with body weight traits. In a study of genetic dissection of growth traits in a Chinese indigenous breed with a commercial broiler cross^81^, the major QTL of body weight was mapped to the end of chromosome 1 (173.7 Mb). Here, associations underlying early growth stages were located on chromosome 1 between 168.74 and 172.99 Mb as indicated by both GLM and MLM analyses. Retinoblastoma 1 (*RB1*) was determined to be the significant gene in this position using a haplotype approach^82^.

Maximum growth rate and body weight at sexual maturity and later ages are similar traits that describe mature body weight and have a high genetic correlation of nearly 1^83^. The sexual maturity in quail is between 6 and 7 weeks of age, while that in chickens is between 16 and 24 weeks of age, depending on the breed^84^. We considered quail’s SNPs associated with body weight around the age of the birds in our study for comparison with the information obtained from chicken studies. The genetic regulation of body weight at maturity and later ages is a multifaceted process involving a network of genes that interact with various physiological systems, hormones, and environmental factors. The complexity of these interactions contributes to the expectation that numerous genes are involved in shaping body weight traits in quails. In a GWAS of carcass traits of Jinghai Yellow chickens^47^, five SNPs were detected for foot weight on chromosome 4 located between 75.54 and 75.67 Mb; thus, indicating that this region is located within 2.08–2.38 Mb away from genes, such as family with sequence similarity 184 member B (*FAM184B*), quinoid dihydropteridine reductase (*QDPR*)*,* and LIM-domain binding factor 2 (*LDB2*). In addition to these genes, Ref.^40^ indicated that the F box and leucine-rich repeat protein 5 (*FBXL5*) has a significant influence on chicken growth traits and important biological functions. They identified this gene to be located on chromosome 4 from 72.9 to 77.9 Mb and to be strongly associated with body weight for weeks 6–16. *QDPR* and *LDB2* were associated with shank circumference^32^ and body weight in Beijing You chickens^38^. Reference^85^ detected two SNPs that were associated with the body weight at the time of oviposition and are located separately at 78.8 Mb and 78.9 Mb on chromosome 4 within the *FAM184B* gene. The non-SMC condensing 1 complex, subunit G (*NCAPG*) gene, which is located within 0.1 kb downstream of the *FAM184B* gene, is a well-known candidate gene for body frame and carcase traits in cattle, and is considered to modulate the body and carcase weight of various breeds^86,87^. At 73.9 and 76.1 Mb, two SNPs associated with ESS_2_ and ESS_1_ on chromosome 4, respectively, were identified by both GLM and MLM analyses. Since ESS is positively correlated with body weight^84^, the influence of these genes on body weight is of considerable importance. Moreover, Ref.^88^ reported the importance of the myostatin (*MSTN*) gene located on chromosome 7 on chicken body weight at 112 days of age. Similarly, we detected numerous associations underlying body weight at maturity and later ages on chromosome 7 at 22.65 Mb in both GLM and MLM analyses. The detection of QTL at later ages has also been reported in chickens^89^. The growth hormone-releasing hormone receptor (*GHRHR*) gene was mapped at position 1.7 cM and polymorphism analysis of this gene revealed three SNPs in the promoter region, which had a significant effect on body weight at 7, 9, 11, 13, and 17 weeks of age^90^. These findings are in line with our SNP association results. In MLM analysis, we identified 10 SNPs that explain over 10% of the phenotypic variance. This underscores their genetic importance and supports our SNP association findings. However, further investigation is needed to uncover the functional roles of these genes, shedding light on their contribution to the observed phenotypic variations at 10–16 weeks of age.

In addition to body weight, egg quality and production traits are the major selection criteria for poultry breeding. The findings of the association study on the TLE and AFE in this study hold significant implications for egg-type poultry breeding. The results have identified specific associations associated with variations in egg production traits, offering valuable insights into the genetic basis of egg production. However, the results of egg production may vary across different studies, and this variation may be attributed to the diverse durations of the egg collection period. In a GWAS investigating egg production and quality traits in chickens, Ref.^91^ found the most significant SNP associated with egg number located within intron 12 of the growth factor receptor-bound protein 14 (*GRB14*) gene. *GRB14* mRNA is highly expressed in the ovary, liver, kidney, and skeletal muscles of humans and mammals^92,93^. Similarly, our MLM suggested that an SNP associated with TLE is located on the same chromosome and position (chromosome 7; 21.46 Mb). It has been speculated that *GRB14* influences egg production in layers^91^. The age of first egg is an important indicator of sexual maturation in female birds and is influenced by genetic and environmental factors^94^. Here, we detected 7 associations that might influence AFE, and they were located on chromosomes 1–4 and Z based on the GLM, and 18 associations positioned on chromosomes 1–3, 5, 8, 12, 19, and 21 based on the MLM analysis. A SNP in intron 2 of the odd oz/ten-m homolog 2 (*ODZ2*) gene has been previously identified to be significantly associated with chicken AFE^91^. This gene is expressed in the developing chicken brain and may affect the sexual maturity^95^. Additionally^96^, demonstrated that the gonadotropin-releasing hormone I (*GnRH-I*) gene is associated with chicken AFE. GnRH stimulates the synthesis and secretion of gonadotrophins, which induce steroidogenesis in the gonads, culminating in ovarian follicle growth and ovulation for egg production^97^. Reference^98^ identified two novel forkhead box L2 (*FOXL2*) and growth differentiation factor-9 (*GDF9*) genes associated with egg production in the Chinese Dagu chickens. These polymorphisms play a critical role in the regulation of ovarian development in hens. This result supports our findings in the present study, in which 53 associations were detected for EPR by both the GLM and MLM analyses.

Our association study focused on external egg quality traits. Eggshell quality is a major concern in poultry breeding due to its implications for both reproductive performance and human consumption. In this study, we successfully identified several associations associated with external egg traits in both egg production stages. These findings shed light on the genetic basis of egg quality and provide valuable information for targeted breeding efforts aimed at enhancing egg production and ensuring high-quality eggs for consumers. A number of QTL regions for egg weight, egg length, and eggshell characteristics have been already identified^17^. In the present study, 49 associations were identified for EW_1,2_ by both the GLM and MLM analyses. The associations for EW on chromosome 4 between 35.49 and 65.59 Mb are in line with the SNP for the same trait and chromosome at 49.28 Mb located in the shroom family member 3 (*SHROOM3*) gene^99^. Shroom3 is a cytoskeletal protein involved in regulating cell shape (arrangement and remodelling) in certain tissues^100^. Among the external egg traits, eggshell characteristics are the most important and play a significant role in the reproductive performance and human consumption. Reference^91^ identified three SNPs for chicken ESW on chromosomes 2 (86.11 Mb), 3 (110.09 Mb), and 11 (9.59 Mb), and two SNPs for EST on chromosome 1 (171.22 and 179.35 Mb). Moreover, they introduced a gene for each detected SNP: polypeptide N-acetylgalactosaminyltransferase 1 (*GALNT1*), *BLK*, zinc finger protein 536 (*ZNF536*), *ENOX1*, and *LOC18918*. These findings support the results of our study, in which two SNPs were detected for ESS_2_ on chromosomes 1 (172.99 Mb) and 11 (14.96 Mb) based on the GLM, and 12 associations were found for ESS_1,2_, ESW_1_, and EET_1,2_ on chromosomes 1 (171.51–174.10 Mb), 2 (84.95–89.81 Mb), and 11 (5.16–14.70 Mb) based on the MLM analysis. The sodium channel (*SCNN1*) gene family is expressed in the active uterus during eggshell mineralisation and plays an essential role in eggshell formation^101^. Polymorphisms of eggshell organic matrix genes were considered to be related to eggshell thickness, eggshell strength, and dynamic stiffness^102^. *SCNN1* has four family members, *SCNN1a*, *SCNN1b*, *SCNN1g*, and *SCNN1d*, which affect eggshell formation^103^. A study investigating eggshell quality traits identified *SCNN1a*, *SCNN1b*, *SCNN1d*, and *SCNN1g* genes on chromosomes 1 (80.03–80.04 Mb), 14 (7.002–7.01 Mb), 21 (2.43–2.44 Mb), and 14 (7.01–7.02 Mb), respectively, and associated them with egg weight, eggshell weight, eggshell percentage, eggshell strength, and eggshell thickness^101^. Our MLM analysis suggested associations for ESS_2_ (chromosome 1, 78.06 Mb), ESW_1_ (chromosome 14, 8.36 Mb), and EW_1_ (chromosome 21, 2.58 Mb), located near the same chromosome, as stated by Ref.^101^. In addition, an association study identified three candidate genes, phosphatidylinositol-4-phosphate 3-kinase catalytic subunit type 2 gamma (*PIK3C2G*), inositol 1,4,5-trisphosphate receptor type 2 (*ITPR2*), and non-SMC condensin I complex subunit G (*NCAPG*) to be implicated in the dynamic eggshell quality and located on chromosomes 1 and 4 from 57.3 to 71.4 Mb^104^. PIK3C2*G* possesses the C2 domain and acts as lipid binding motif, *ITPR2* has been shown to be important to the process of eggshell calcification, and *NCAPG* gene was discovered to be associated with eggshell weight for young hens in a genome-wide association analysis^104^. Moreover, one association analysis determined that another lipid-related gene, low-density lipoprotein receptor-related protein 8 (*LRP8*), a new member of the egg shell matrix protein family, was significantly associated with eggshell traits^105^. Our MLM findings are in support of the aforementioned studies, in which 6 associations were identified on chromosome 1 between 60.20 and 67.94 Mb for EW_2_ and 5 associations were detected for EW_1_, ESS_2_, and ESW_1_ on chromosome 4 between 58.94 and 69.85 Mb.

In our egg quality association study, we extended our investigation to internal aspects, such as yolk weight, diameter, color, and albumen weight. Understanding the genetic associations influencing internal egg quality is crucial due to the shift towards egg products in consumption. Through genetic marker analysis, we identified significant associations in internal egg characteristics, providing valuable insights for targeted breeding to enhance yolk weight and meet consumer preferences in the egg industry. Reference^91^ identified the ataxia telangiectasia mutated (*ATM*) gene located on chromosome 1 (184.63 Mb) to be associated with the YW trait. In the present study, based on MLM analysis, we introduced 17 associations for YW_1,2_ on chromosome 1 between 67.94 and 172.99 Mb. Cathepsin D (*CTSD*) is another candidate gene that might affect egg characteristics^106^. Yolk formation involves cholesterol uptake and transport mediated by the very-low-density lipoprotein receptor on the membrane, and *CTSD* is the key enzyme regulating this process^107^. In this study, the detection of a wide range of associations (354) on all chromosomes (except for 22, 24, and 28) suggests that *CTSD* is a candidate gene associated with egg yolk quality traits. The albumen makes up approximately two-thirds of an egg’s weight. Numerous QTLs have been reported for egg white characteristics according to the chicken QTL database (https://www.animalgenome.org/cgi-bin/QTLdb/GG/index). Two promising genes, dopamine receptor D1 (*DRD*1) and msh homeobox 2 (*MSX2*) were found to be associated with albumen characteristics^108^. *DRD1* encodes the D1 subtype of the dopamine receptor^109^. In birds, dopamine has been manifested to be involved in both stimulating and inhibiting prolactin (PRL) secretion, which has been illustrated to play an important role in the onset and maintenance of incubation behaviour^110^. Moreover, it has been shown to be associated with egg production traits^109^. MSX2 is a member of the msh homeobox family and is present in various embryonic tissues^108^. In the developing chick, *MSX2* is expressed in the apical ectodermal ridge and the ectoderm of the genital tubercle; thus, playing a crucial role in the growth and patterning of the limb mesoderm^111^. Since *MSX2* and *DRD1* are involved in embryonic and ovarian development^108^, they could be treated as candidate genes associated with egg quality. Here, 23 associations were discovered for AW_1,2_ located on chromosomes 1–3, 8–9, 14, 21, 23, and Z in the GLM and MLM analyses. Yolk colours are important internal egg traits that are influenced by genetic and environmental factors, most often through the components which are included in the quail’s diet^112^. A total of 231 associations were found in the present study for SNPs that might influence yolk colours (L*, a*, and b* values) in both the GLM and MLM analyses. These associations were located on all chromosomes except for chromosomes 13, 22, 24, and 28. Genes underlying yolk colours have been previously reported in Galliformes. Reference^113^ introduced three candidate genes, glucagon-like peptide 1 receptor (*GLP1R*), calcitonin receptor (*CALCR*), and tissue factor pathway inhibitor 2 (*TFPI2*), and suggested they are significantly associated with yolk colour. *GLP1R* is a member of the glucagon receptor family of G protein-coupled receptors found on beta cells in the pancreas and on neurons, and is involved in controlling blood sugar levels^114^. *CALCR* encodes a calcitonin receptor whose activity is mediated by G proteins which activate adenylyl-cyclase and is involved in calcium homeostasis, bone formation and metabolism, as well as lipid metabolism^115^. *TFPI2* is a Kunitz-type serine protease inhibitor that inhibits matrix metalloproteinase activation and extracellular matrix degradation^116^. These genes are located on chromosome 5 within the 21.06–21.36 Mb genomic region^113^. In the present study, based on the MLM analysis, the nearest located SNP associations were in the 18.53–25.60 Mb range on chromosome 5 and responsible for YC-L*_1_ and YC-b*_2_ (Supplementary File S3). This similarity in positional candidate genes may indicate that our associations control yolk colour characteristics.

In this study, we applied the Bonferroni-corrected GLM and MLM to investigate the genetic basis of targeted traits. Our R^2^ values conform to established statistical practices, considering multiple comparisons. The variability in R^2^ values can be attributed to trait complexity and genetic diversity. Comparative analysis with existing research supports the significance of our findings, contributing to the understanding of targeted traits.

By the assessment of 44 phenotypic traits using GLM and MLM approaches, we showed the existence of SNP associations with all targeted traits, with the exception of EW_1_, AW_1_, and YC-b*_2_ in the GLM and ELA_2_ in the MLM analysis. The observed differences in GLM and MLM results may be influenced by factors like population structure, handling of missing data, genetic architecture, and assumptions made by each model. In agreement with previous studies, we hypothesize that multiple positional candidate genes are associated with body weight and egg-related traits. Future studies focusing on genotyping the SNPs within these candidate genes or linked markers would be of great use to improve the associations at the gene level.

To the best of our knowledge, this is the first study to perform an association analysis of the production traits of Japanese quail. A total of 383 SNPs and 1151 associations were obtained following GLM, and 734 SNPs and 1442 associations were obtained following MLM analyses. The identified associations were significantly correlated with body weight and egg production. Moreover, 35 annotated genes were introduced as candidate genes for the targeted traits based on their nearest positions. Identifying associations and candidate genes could contribute to better understanding the genetic factors controlling body weight, egg production, and quality traits in quails particularly, and chickens generally, and may accelerate the genetic progress in breeding strategies.

### Supplementary Information

Below is the link to the electronic supplementary material.

Supplementary Information 1.

Supplementary Information 2.

Supplementary Information 3.

Supplementary Information 4.

Supplementary Information 5.

## Data Availability

RAD-seq read data were deposited in the DDBJ Sequence Read Archive (accession no. DRA011153) https://ddbj.nig.ac.jp/search.
