# Supplementary material for: Association analysis of production traits of Japanese quail (Coturnix japonica) using restriction-site associated DNA sequencing
Source: Sci Rep. 2023 Dec 2;13:21307. doi: 10.1038/s41598-023-48293-0 (PMC10693557; doi:10.1038/s41598-023-48293-0)
Supplement: Supplementary file 4 — Supplementary Information 4. [file 41598_2023_48293_MOESM4_ESM.docx]

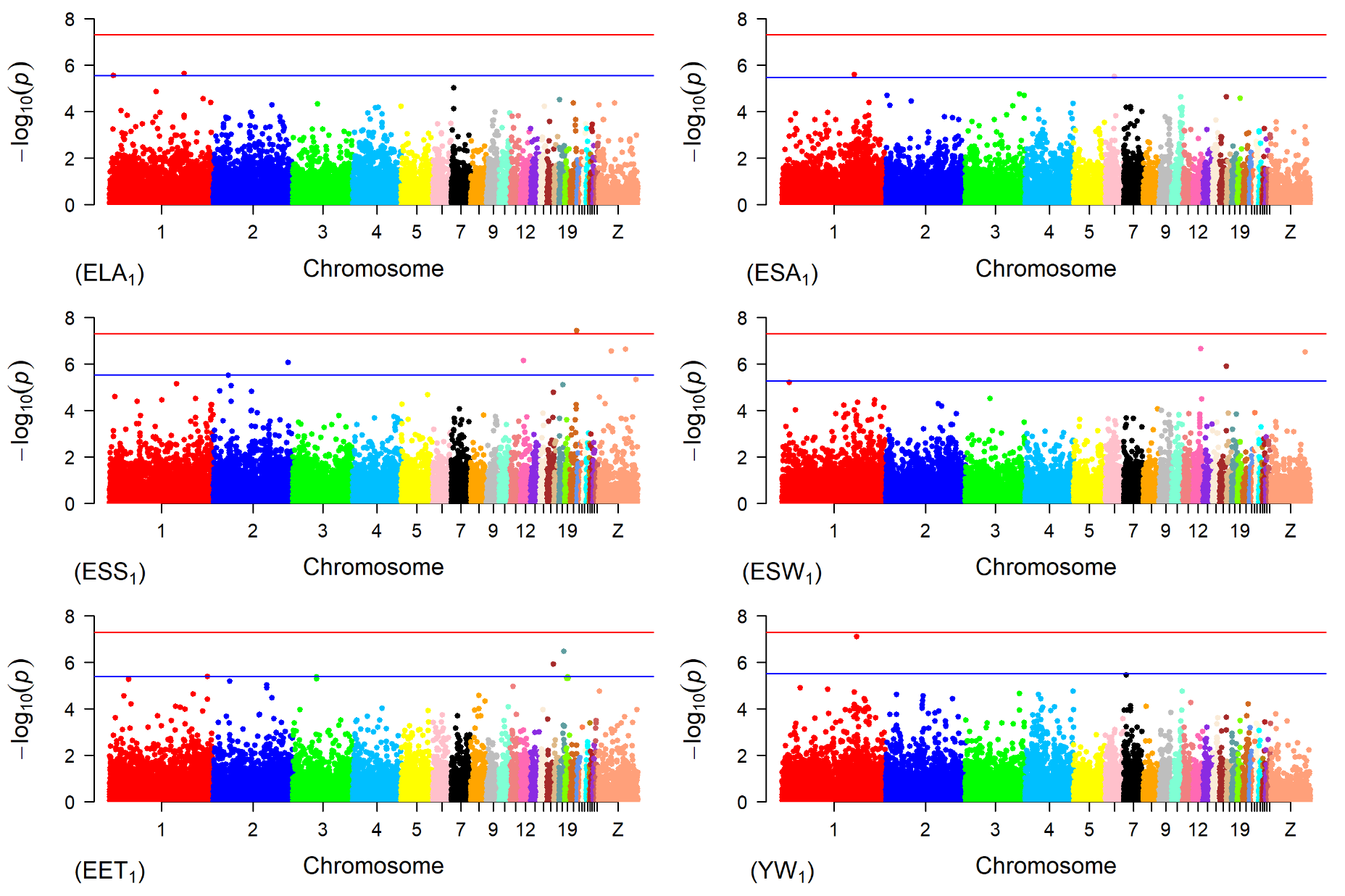


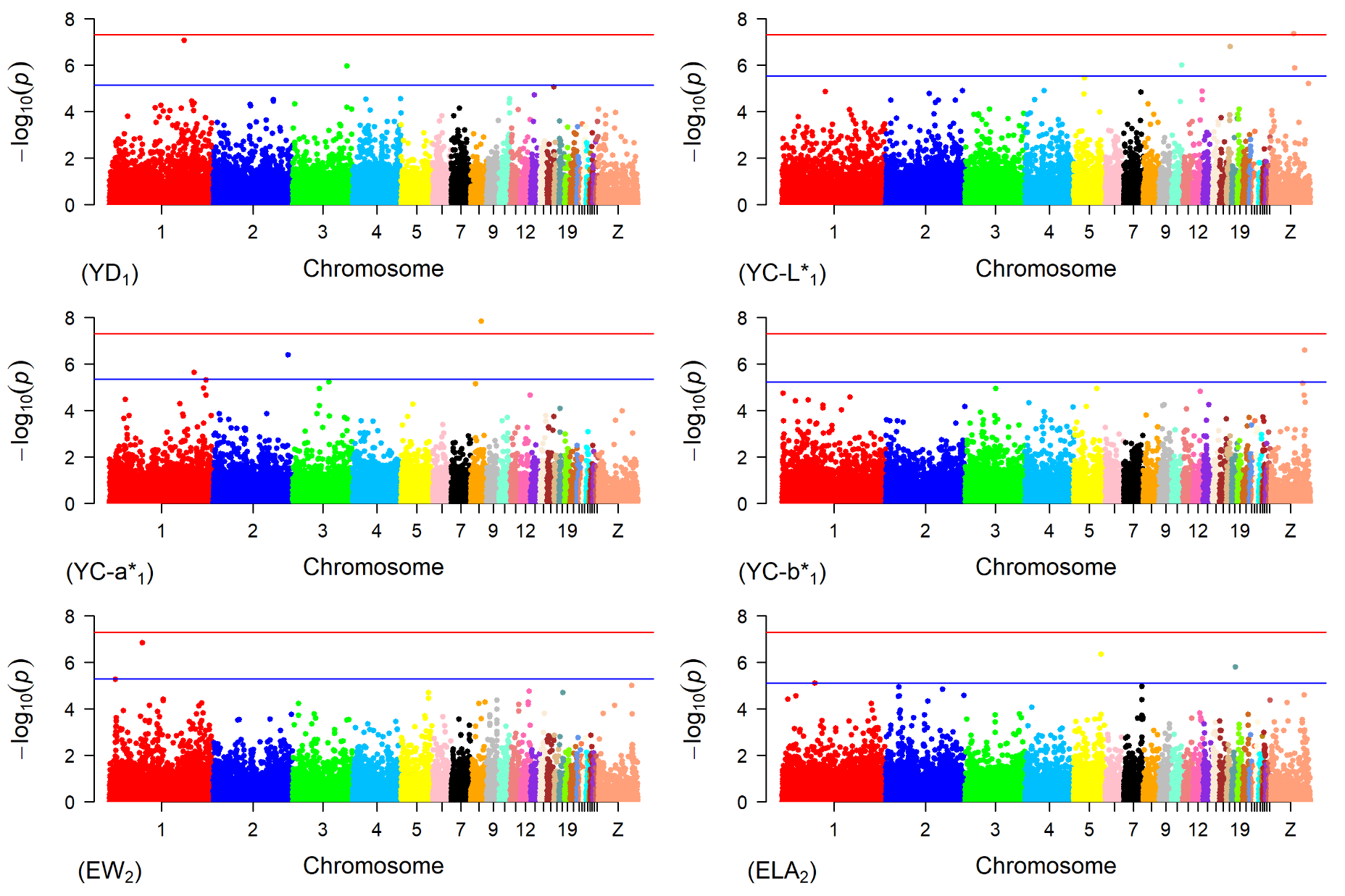


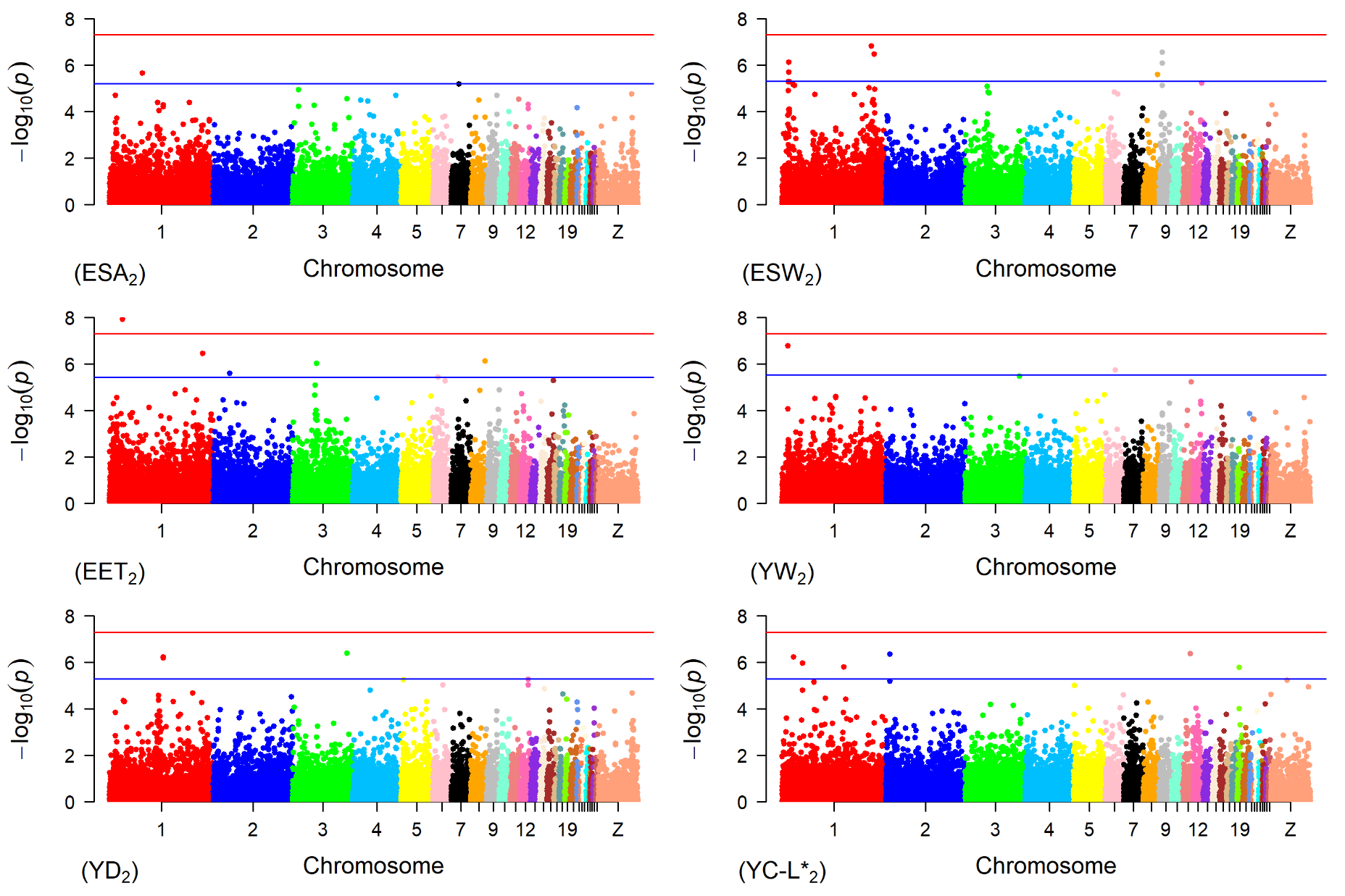


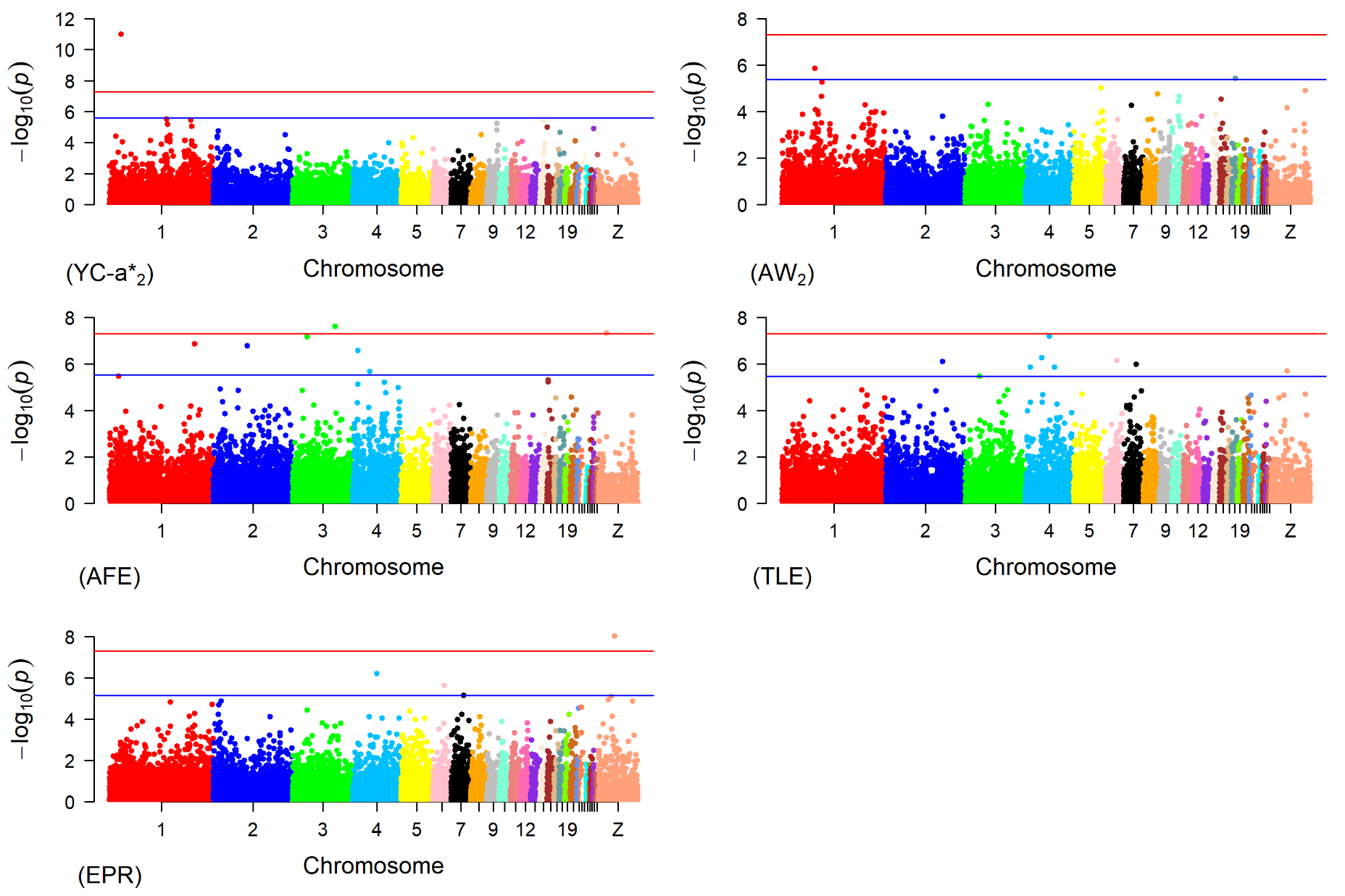


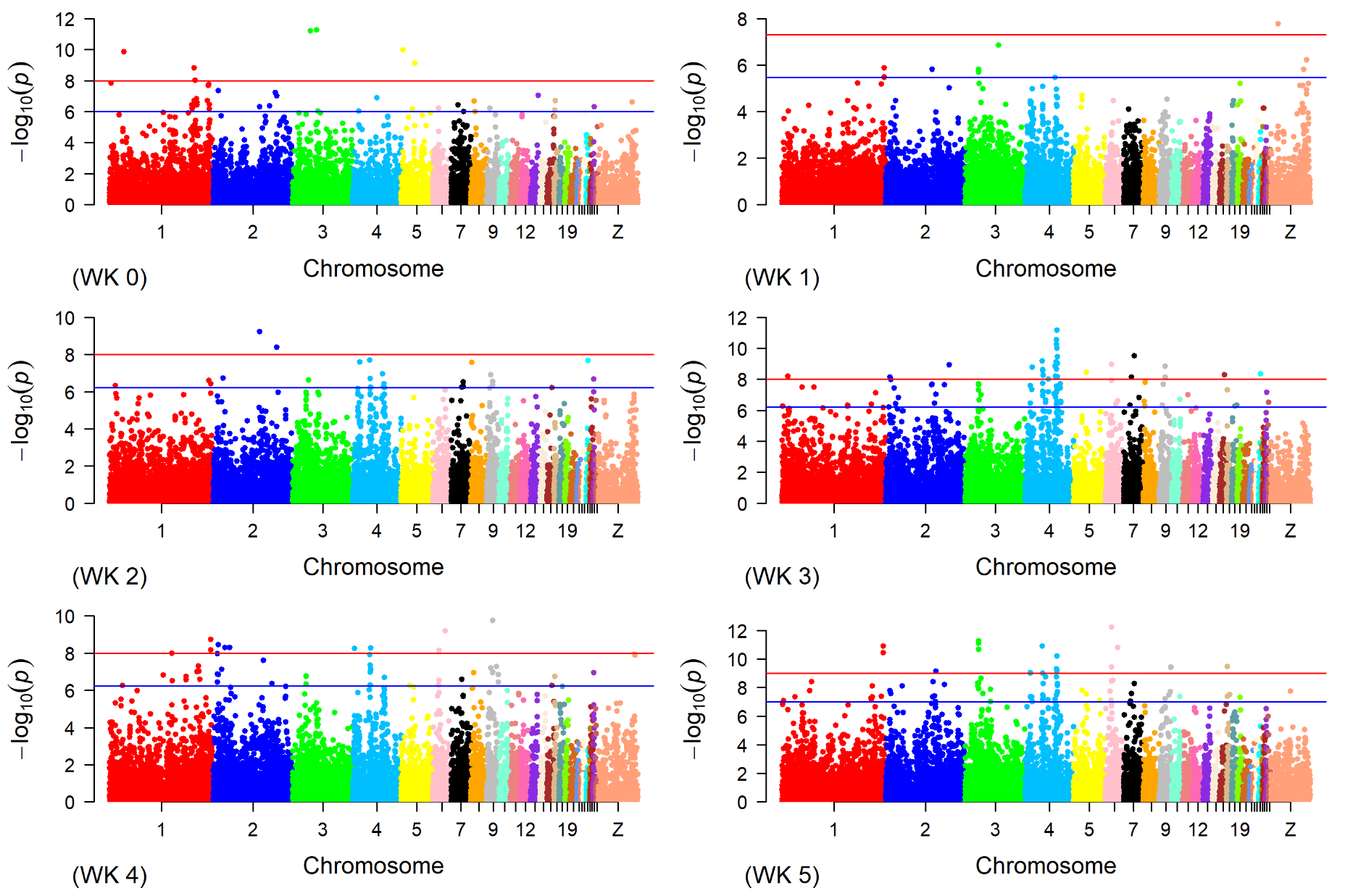


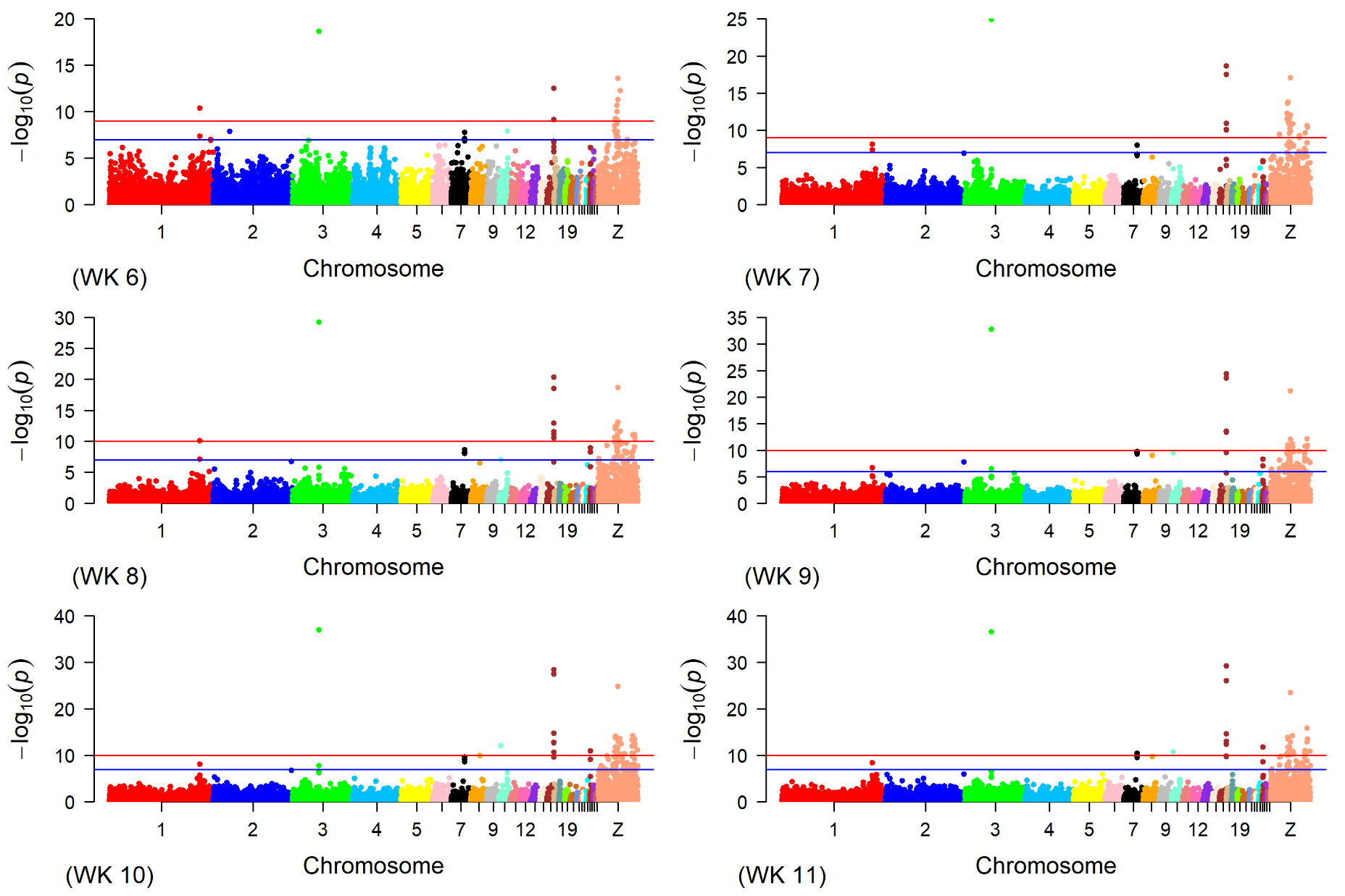


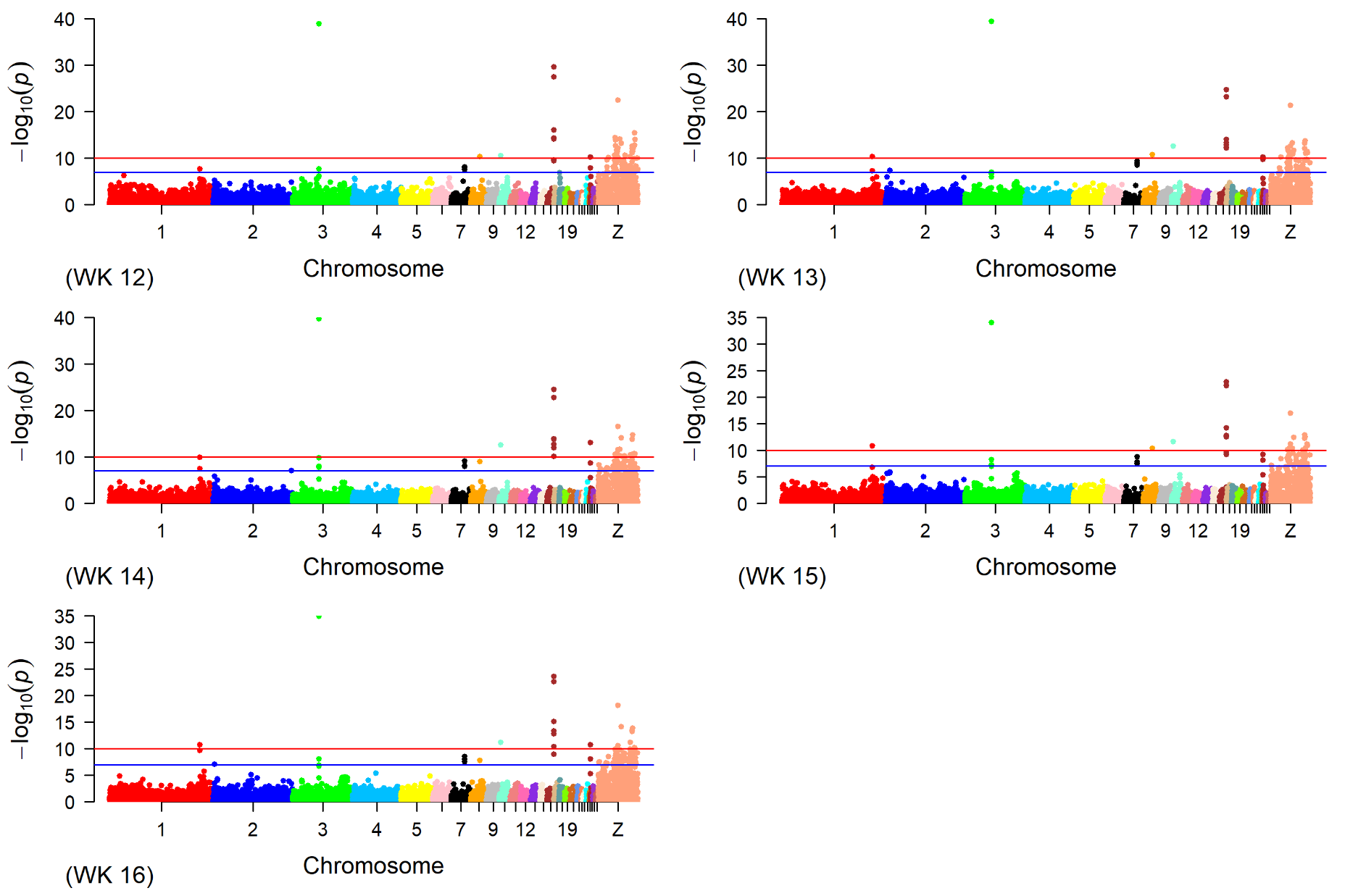


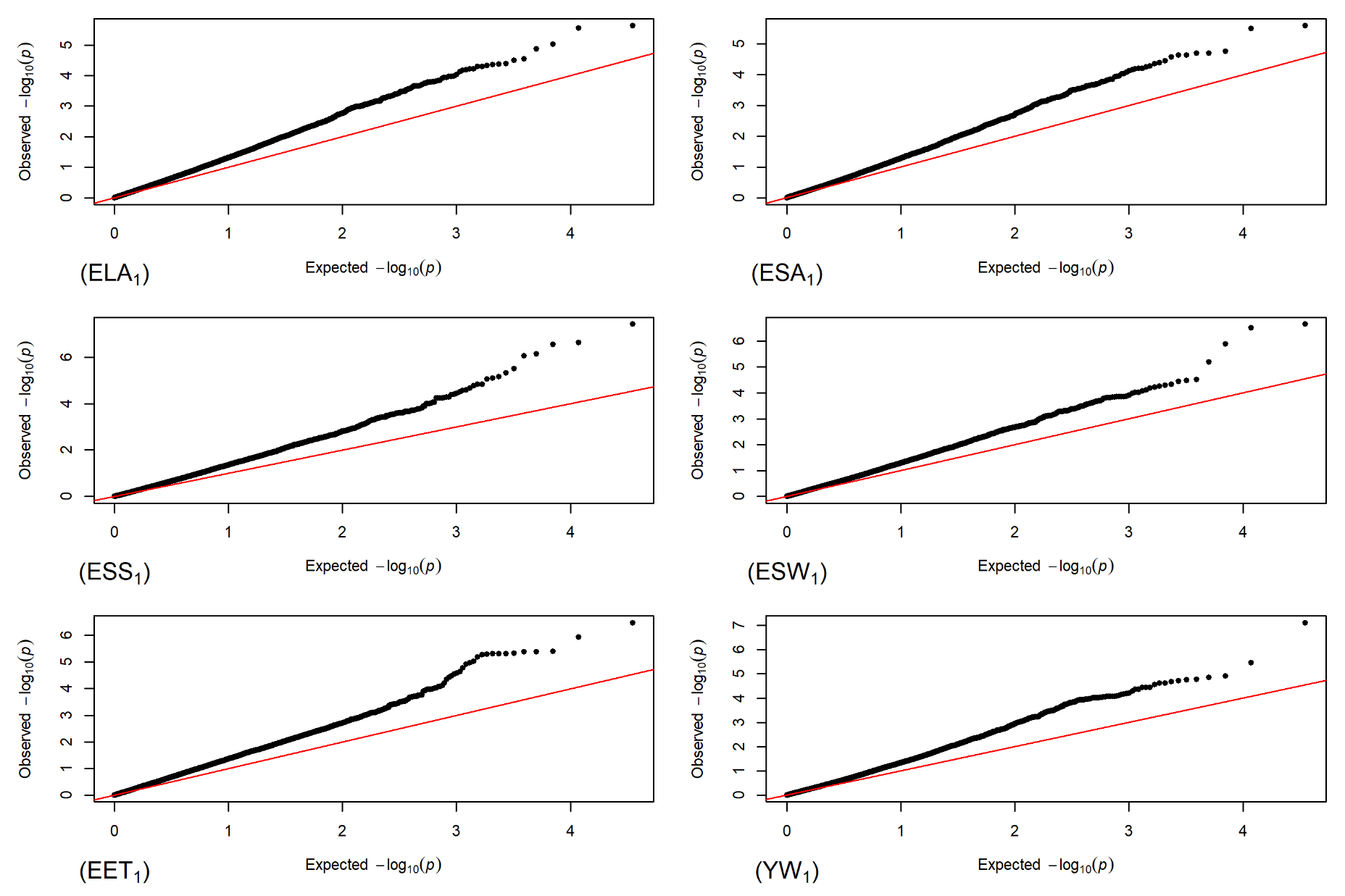


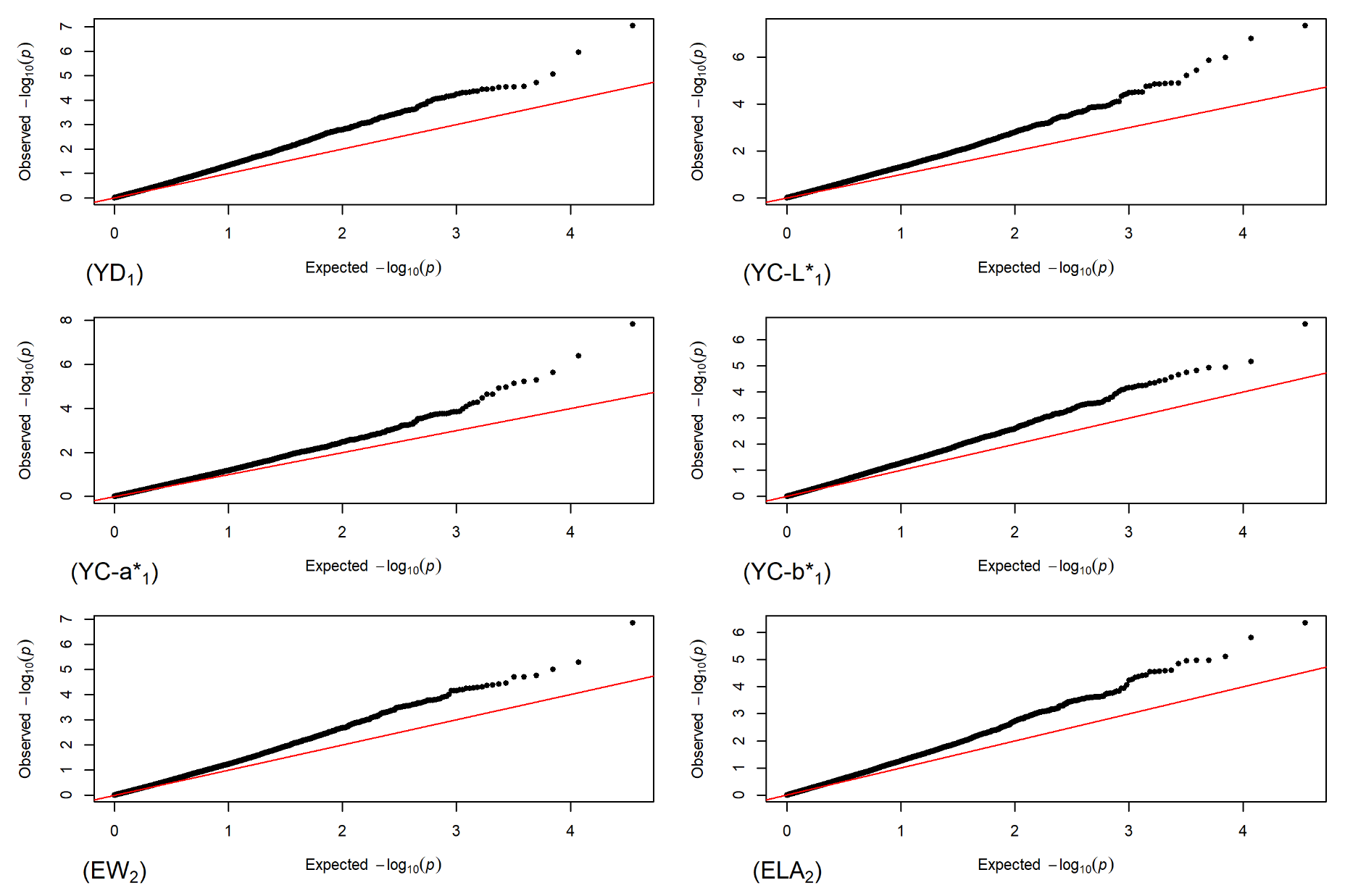


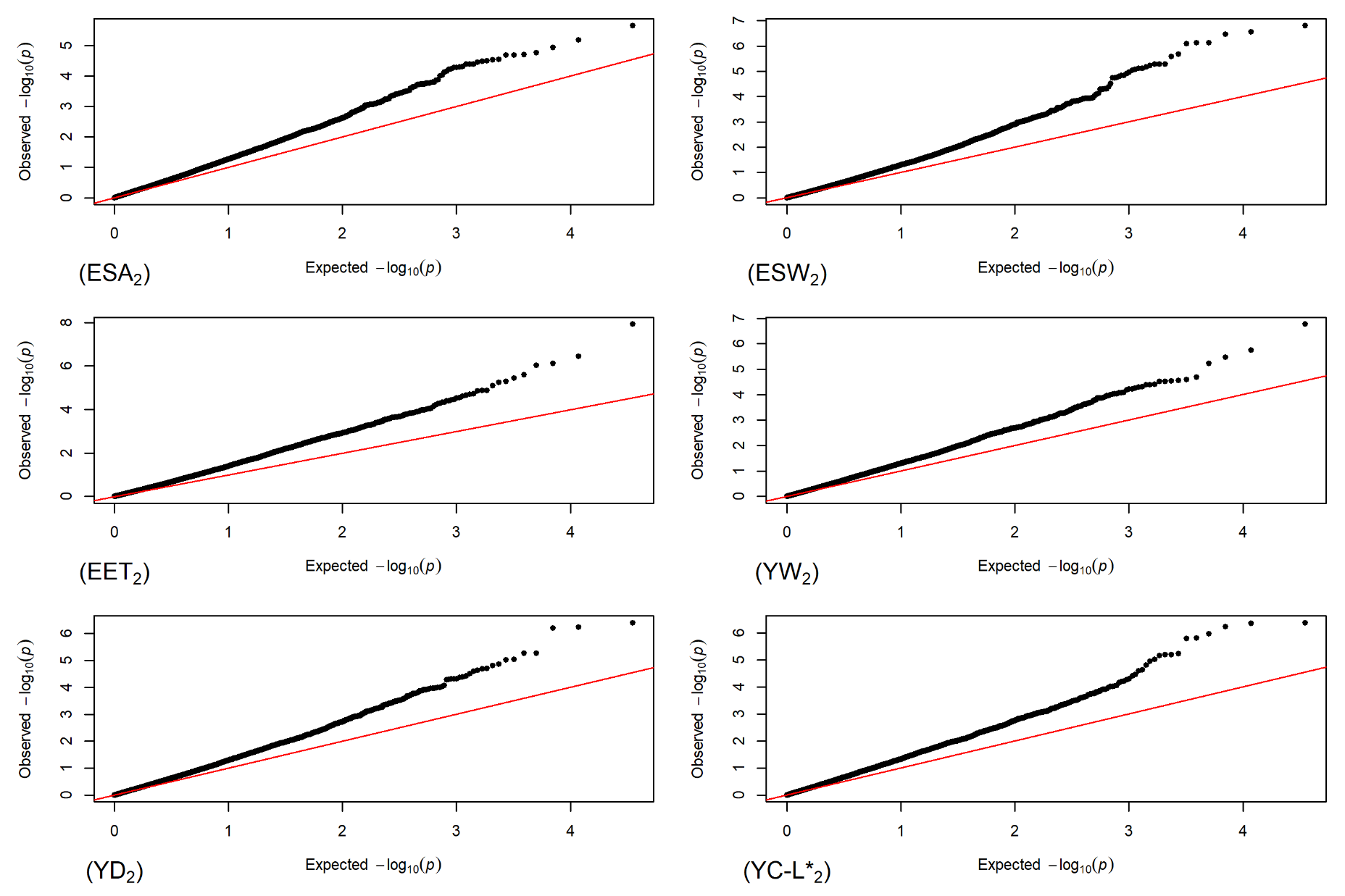


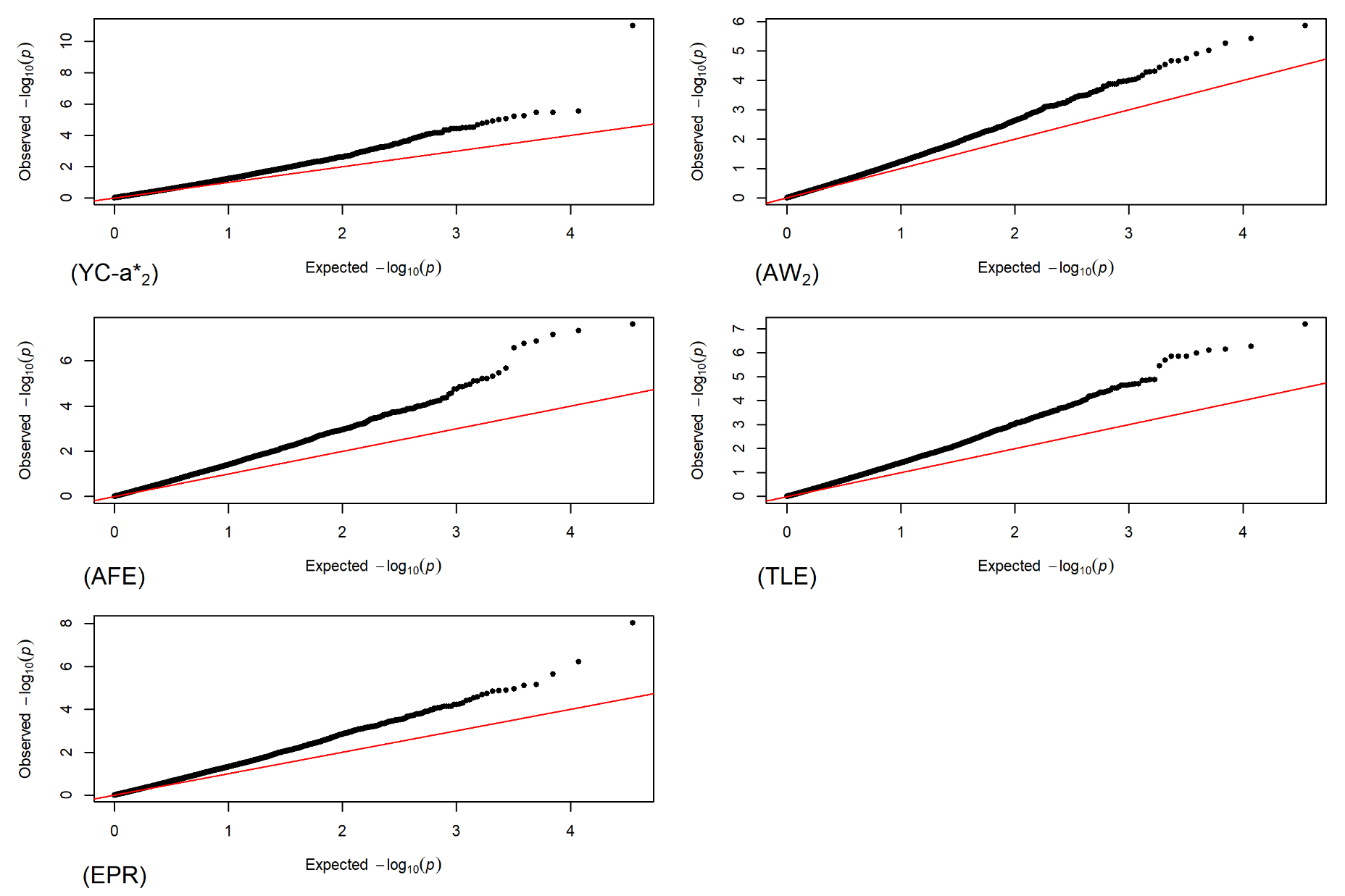


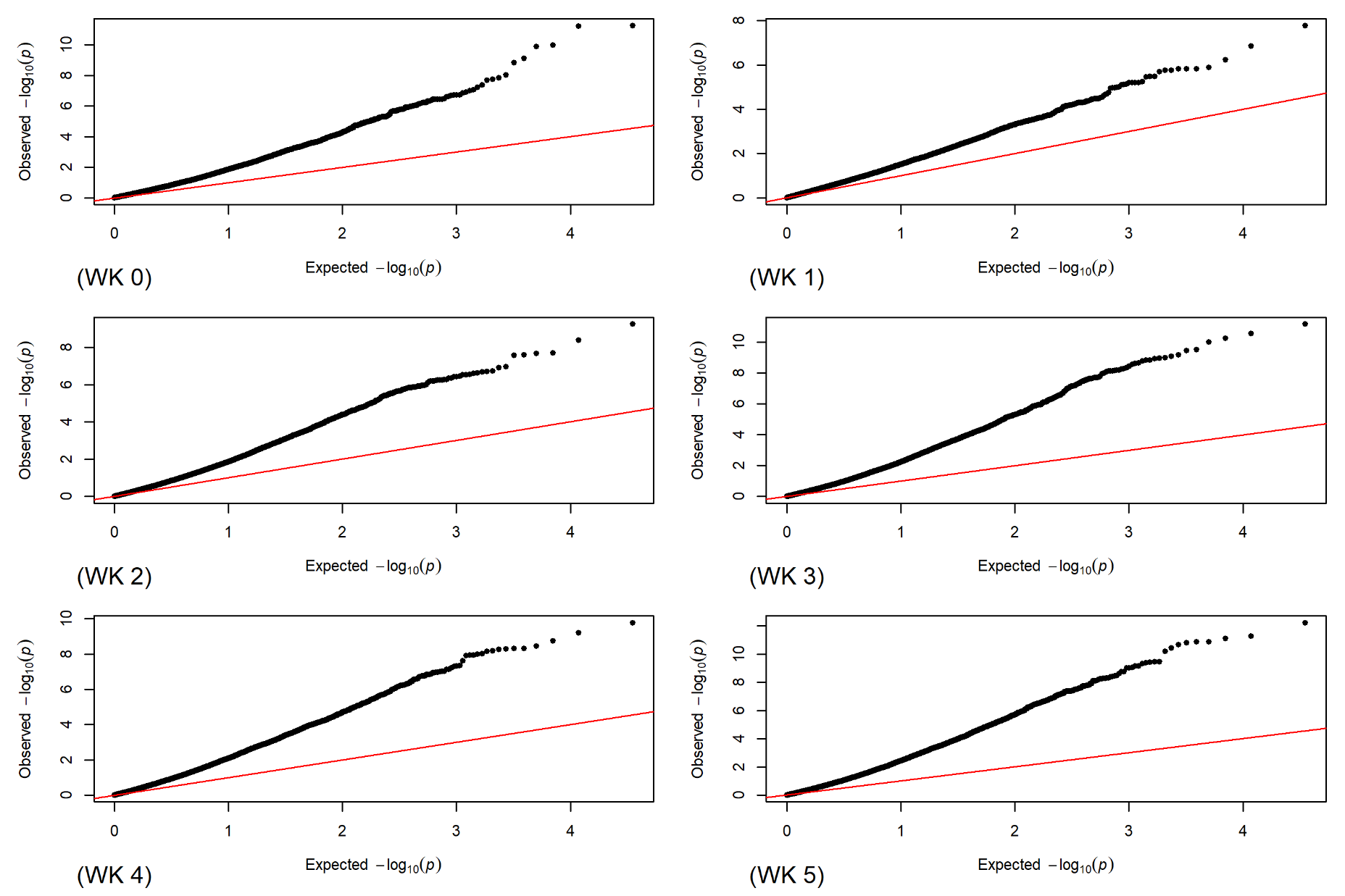


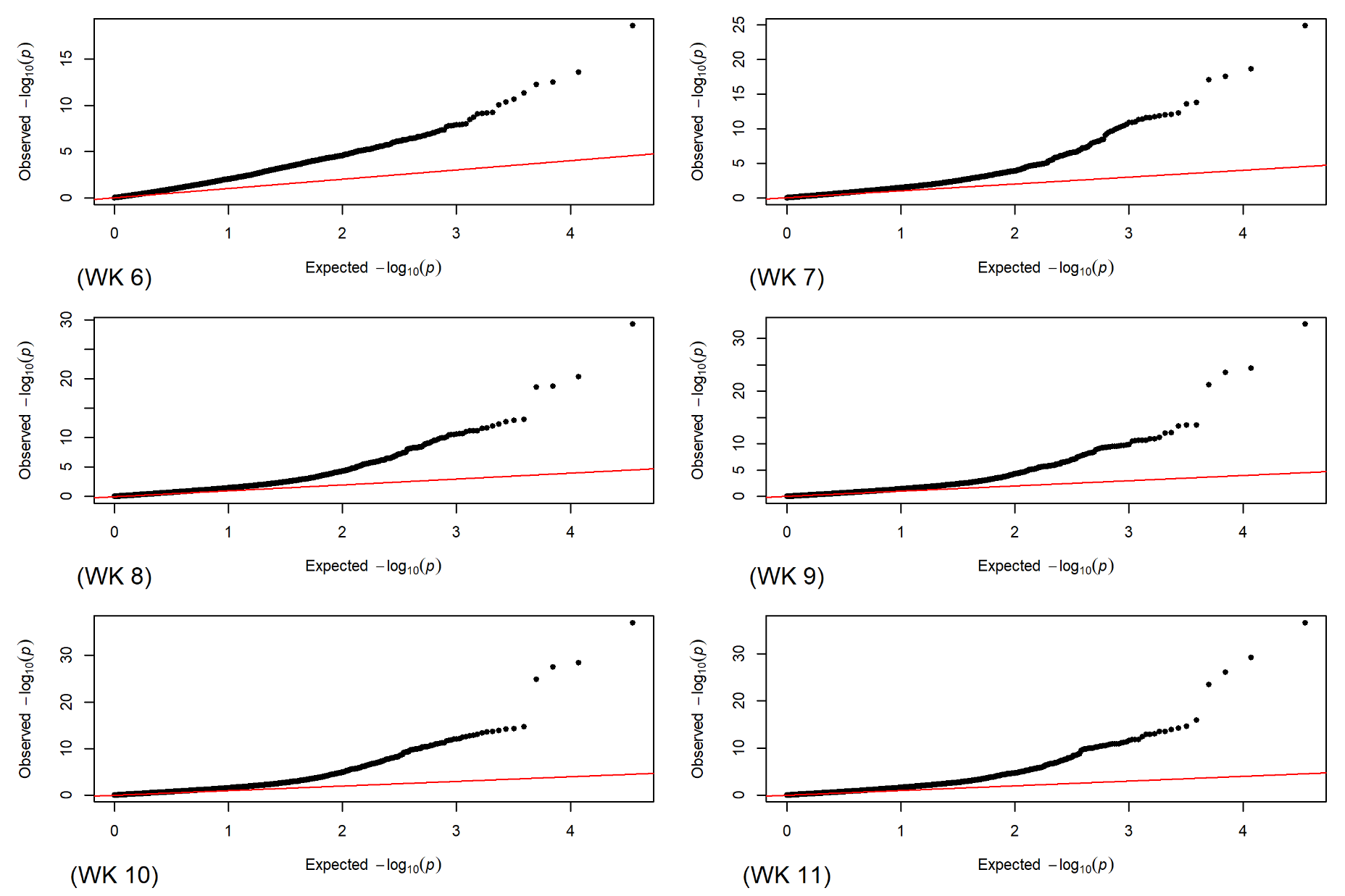


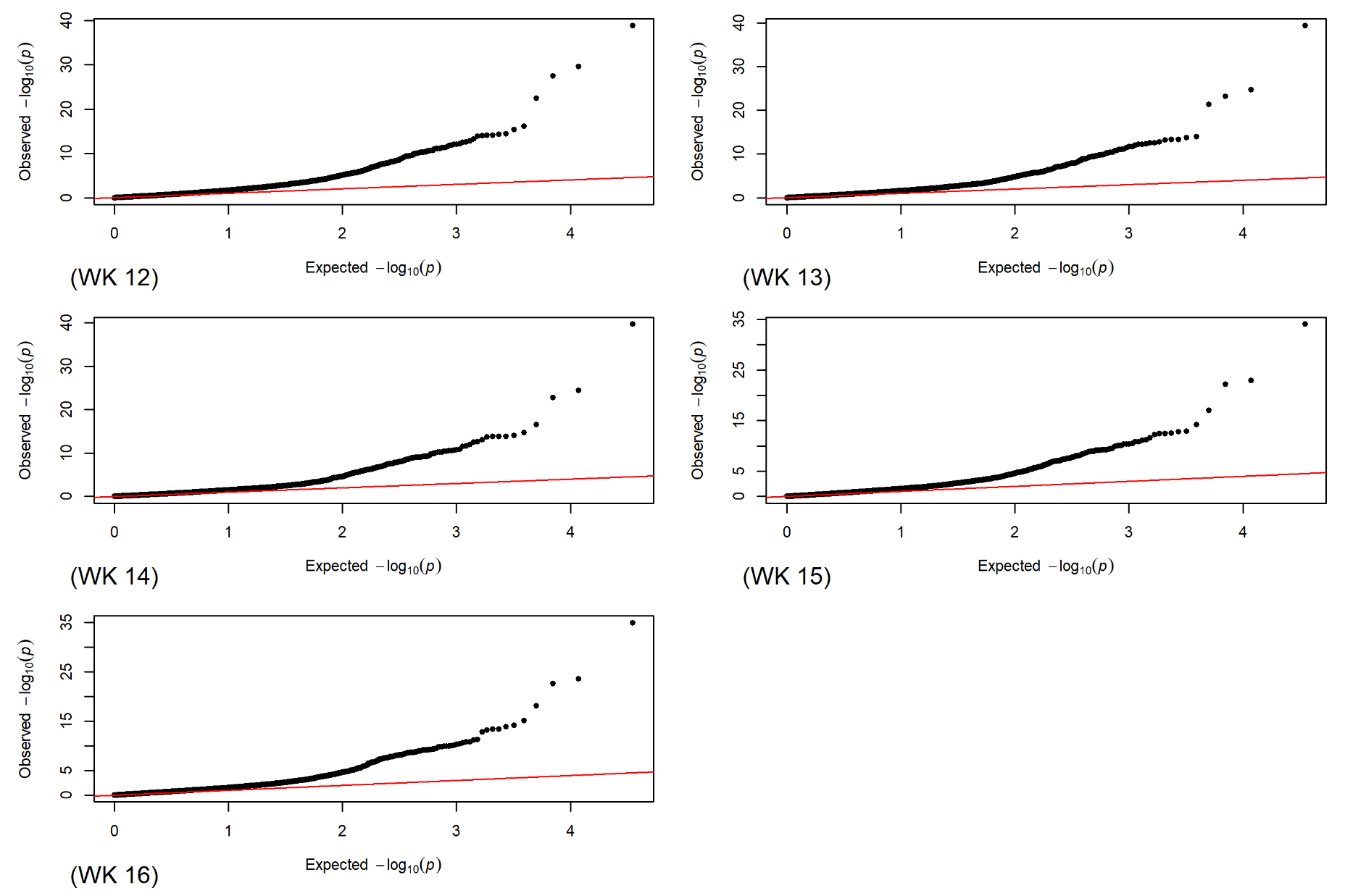


**Supplementary File 4**. The Manhattan and QQ plots for all traits (except for ESS_2_) using the GLM approach. In Manhattan plots, each dot represents an SNP. The figure illustrates the level of statistical significance (y-axis) as measured by the negative log of the corresponding *p*-value for each SNP. Each SNPs type is indicated by dots of different colours, which are arranged by chromosomal location (x-axis). The horizontal red line indicates the threshold of 5% Bonferroni genome-wide significance, and the blue line presents a genome-wide suggestive.
