# Supplementary material for: Association analysis of production traits of Japanese quail (Coturnix japonica) using restriction-site associated DNA sequencing
Source: Sci Rep. 2023 Dec 2;13:21307. doi: 10.1038/s41598-023-48293-0 (PMC10693557; doi:10.1038/s41598-023-48293-0)
Supplement: Supplementary file 5 — Supplementary Information 5. [file 41598_2023_48293_MOESM5_ESM.docx]

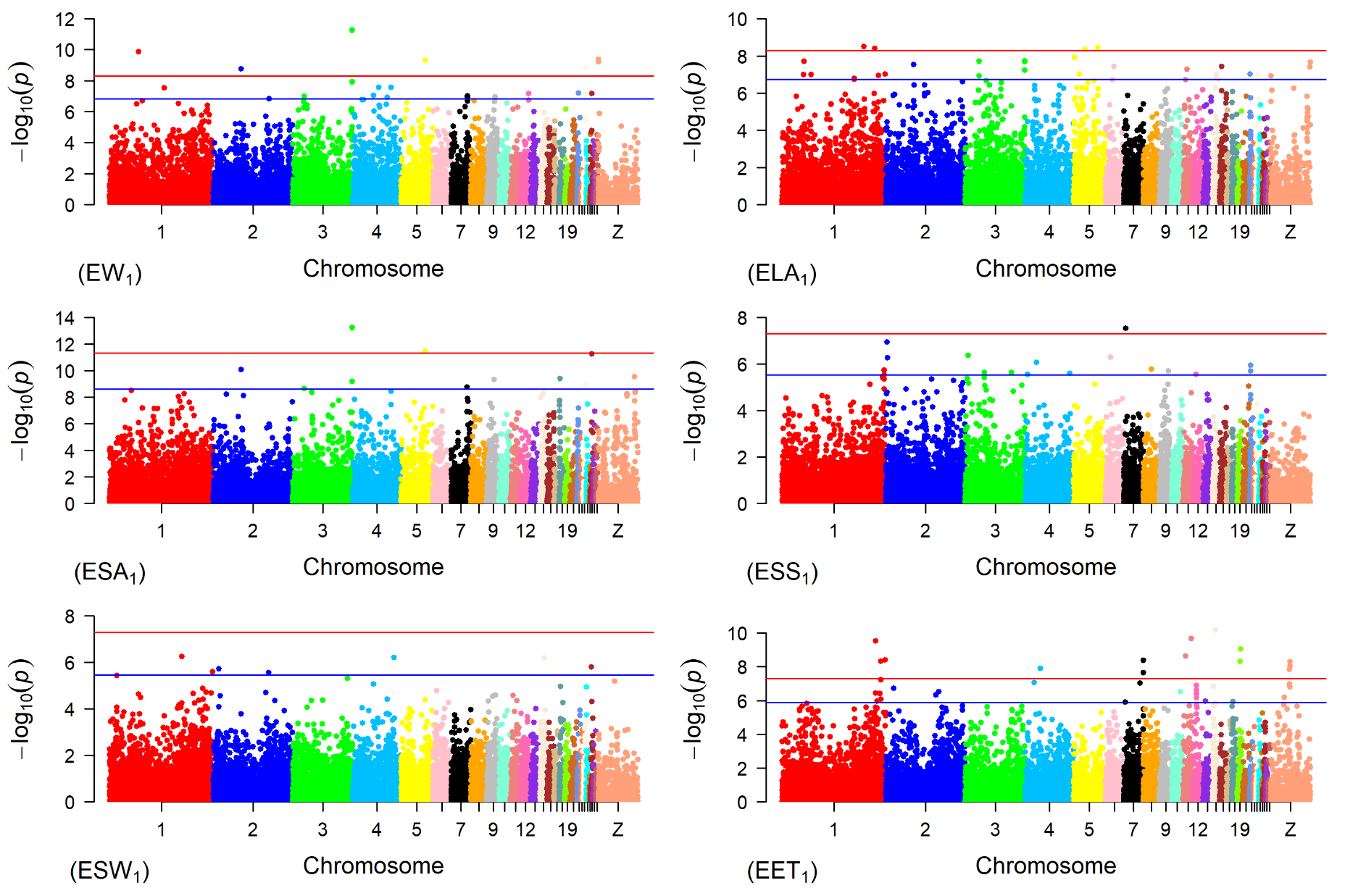


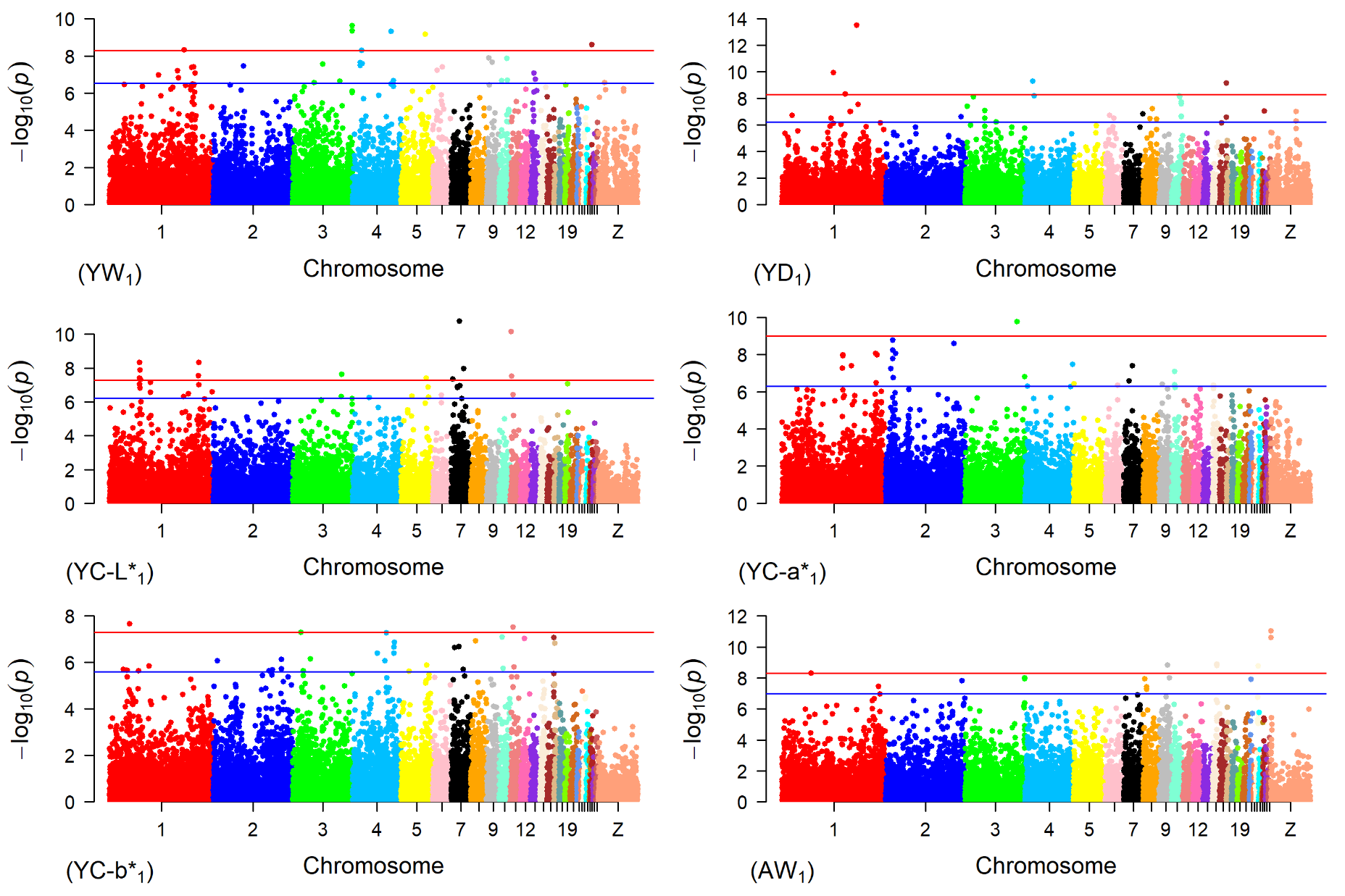


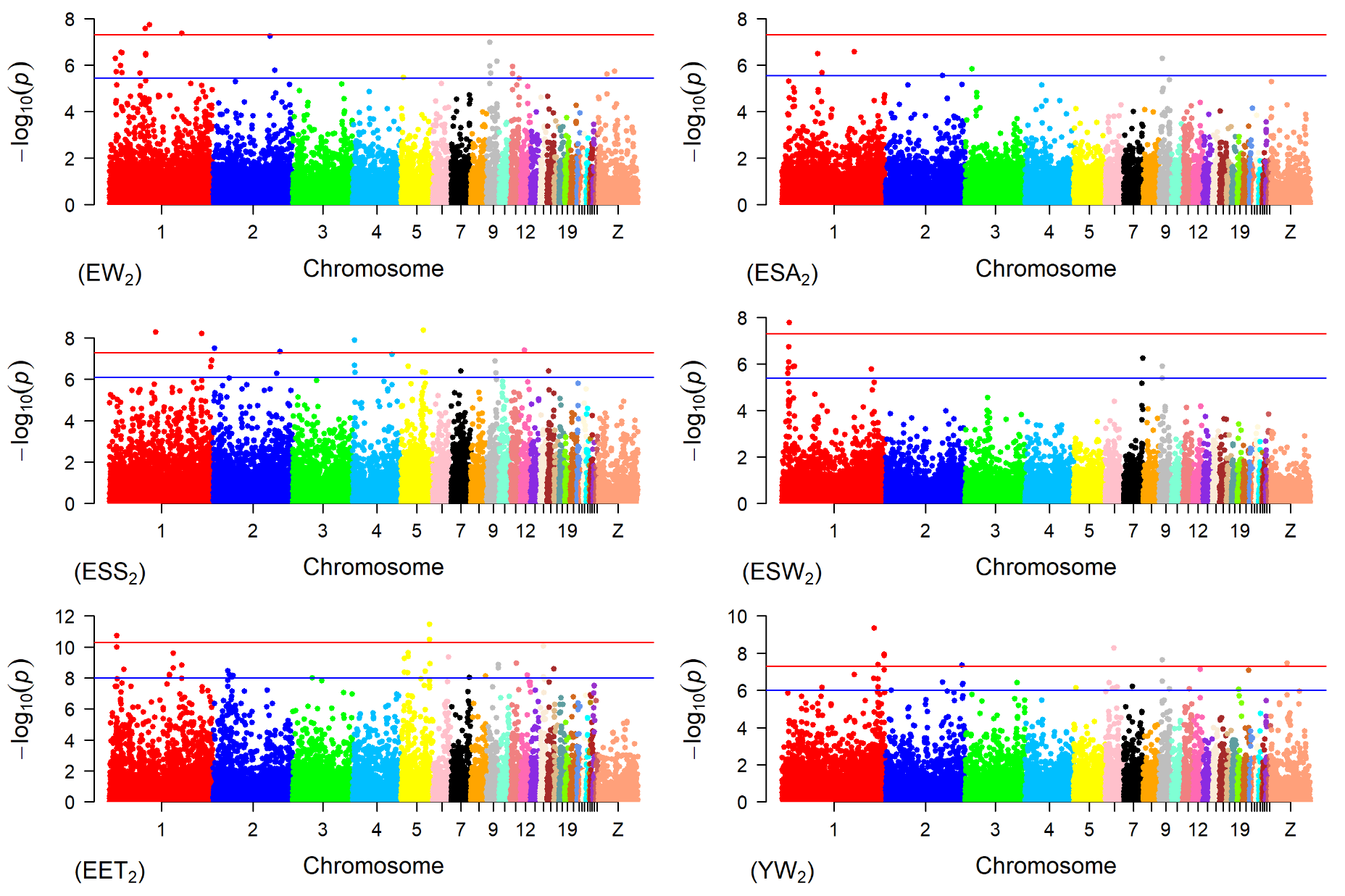


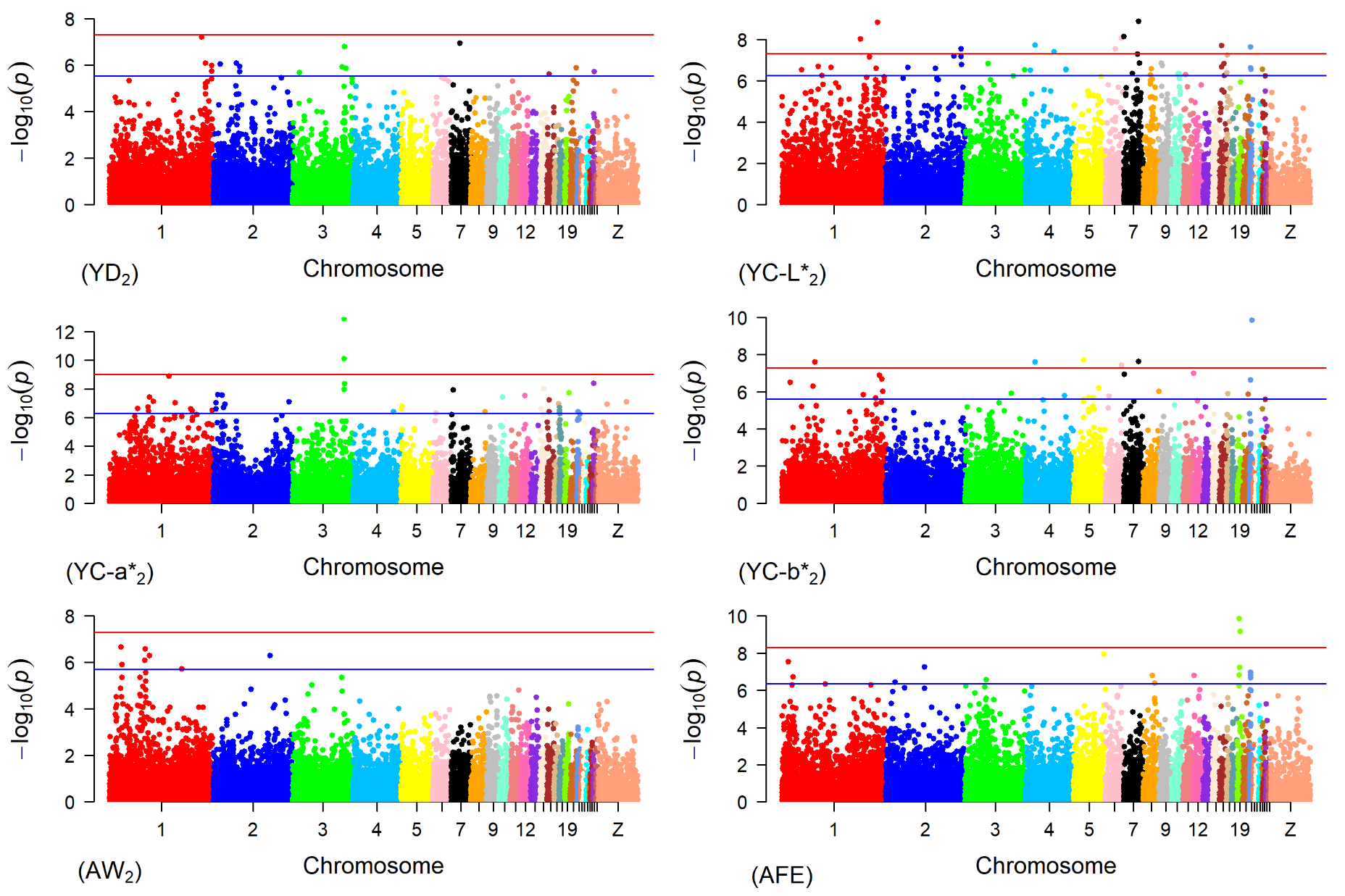


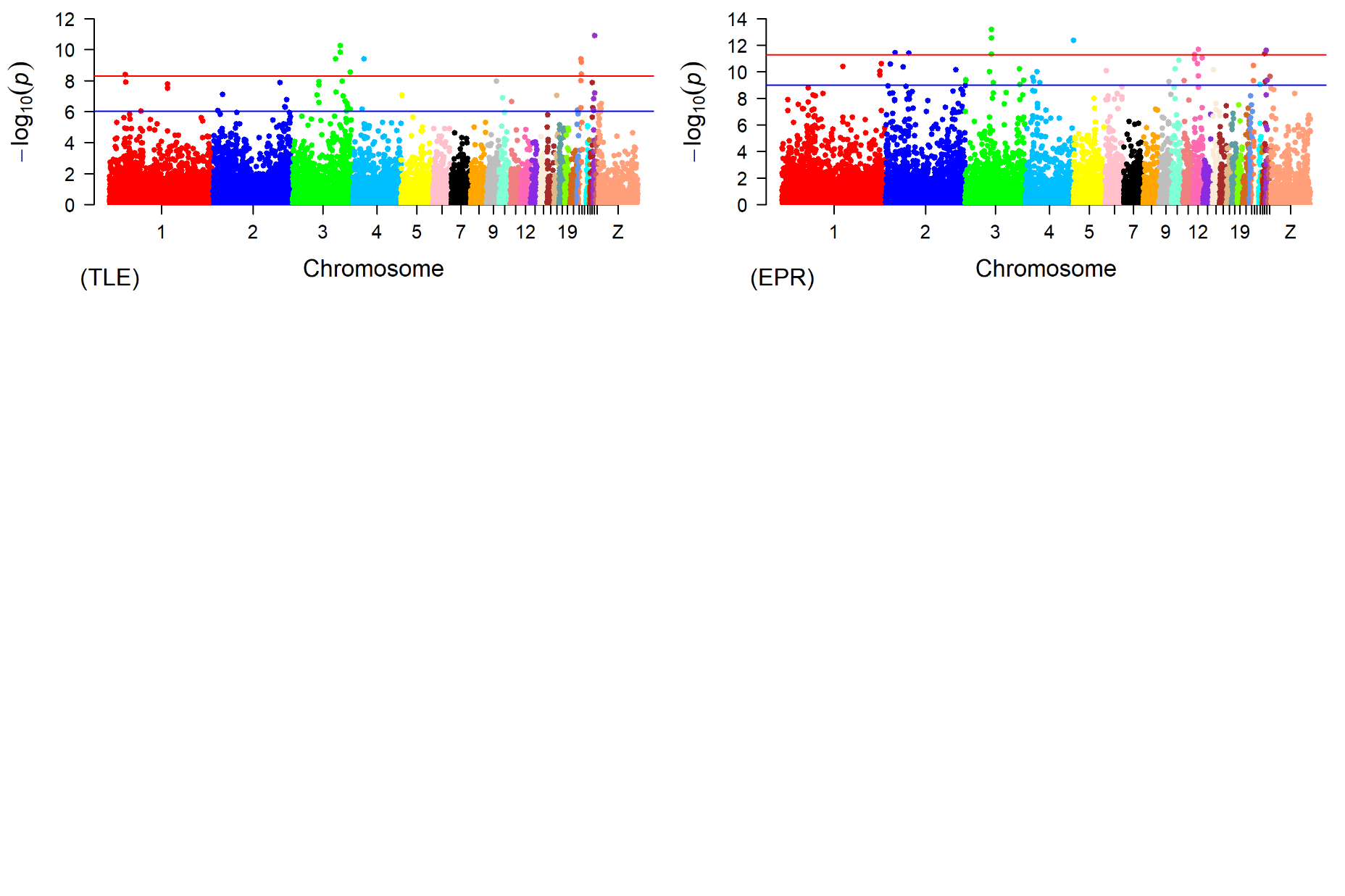


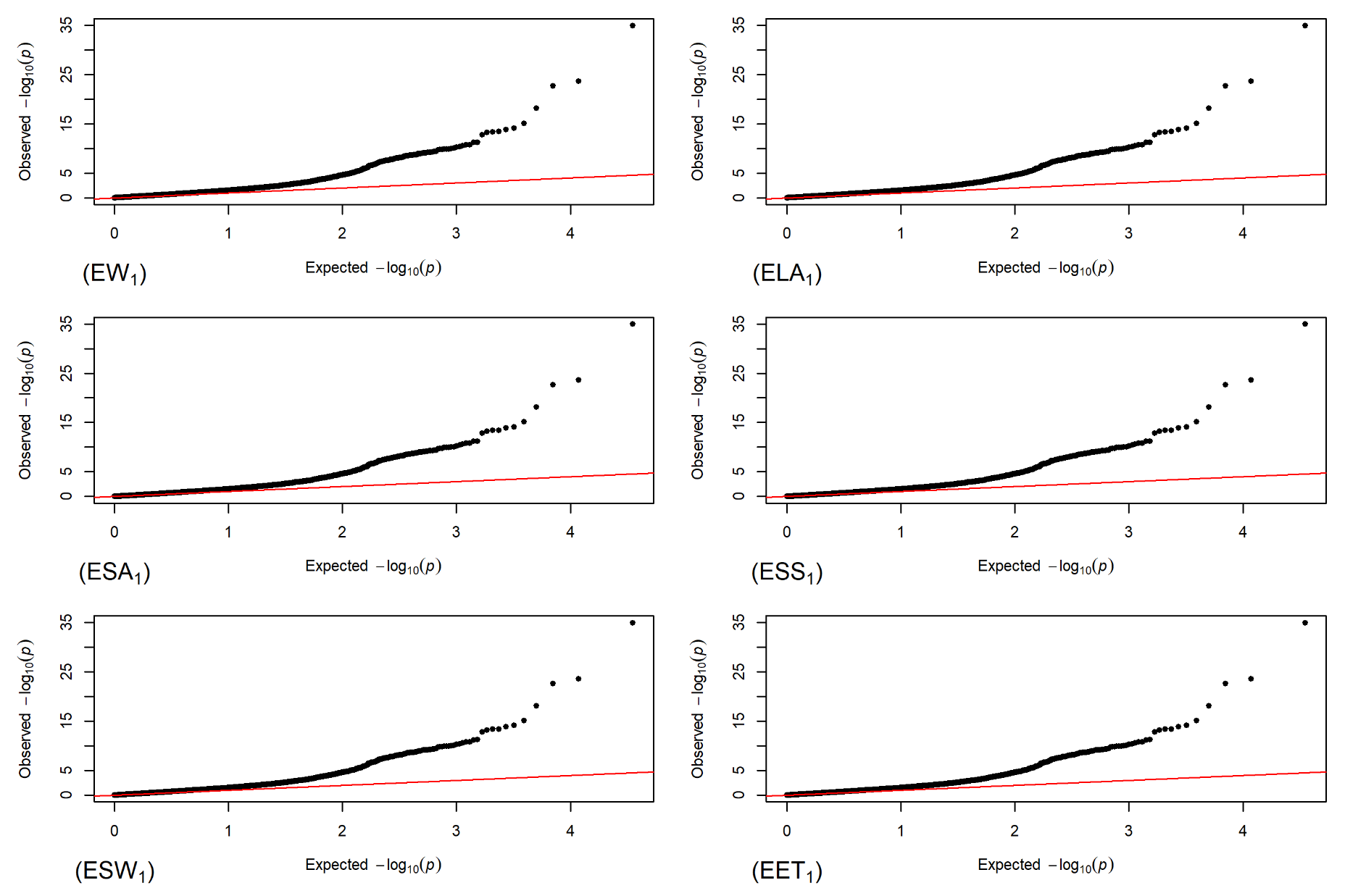


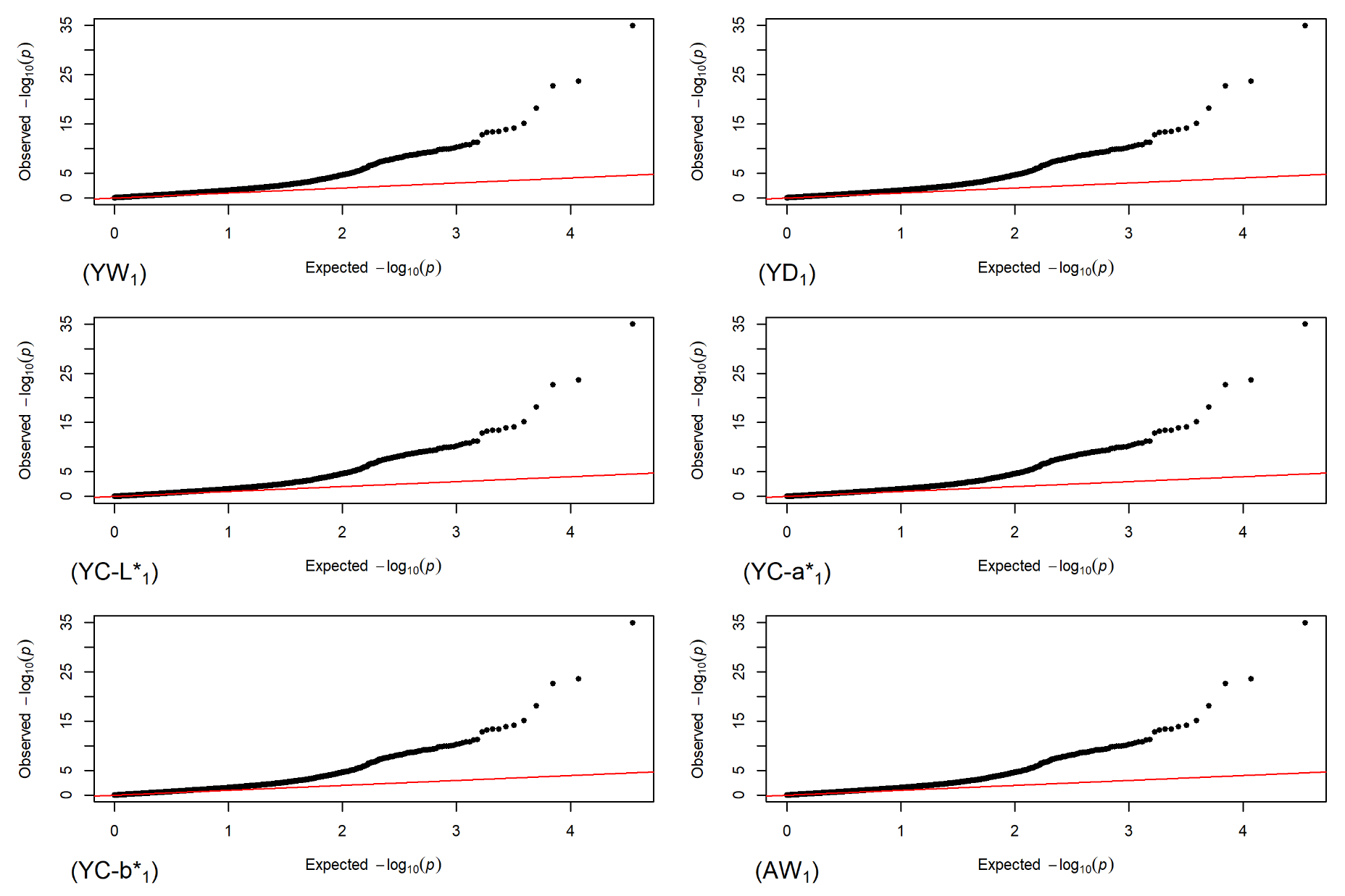


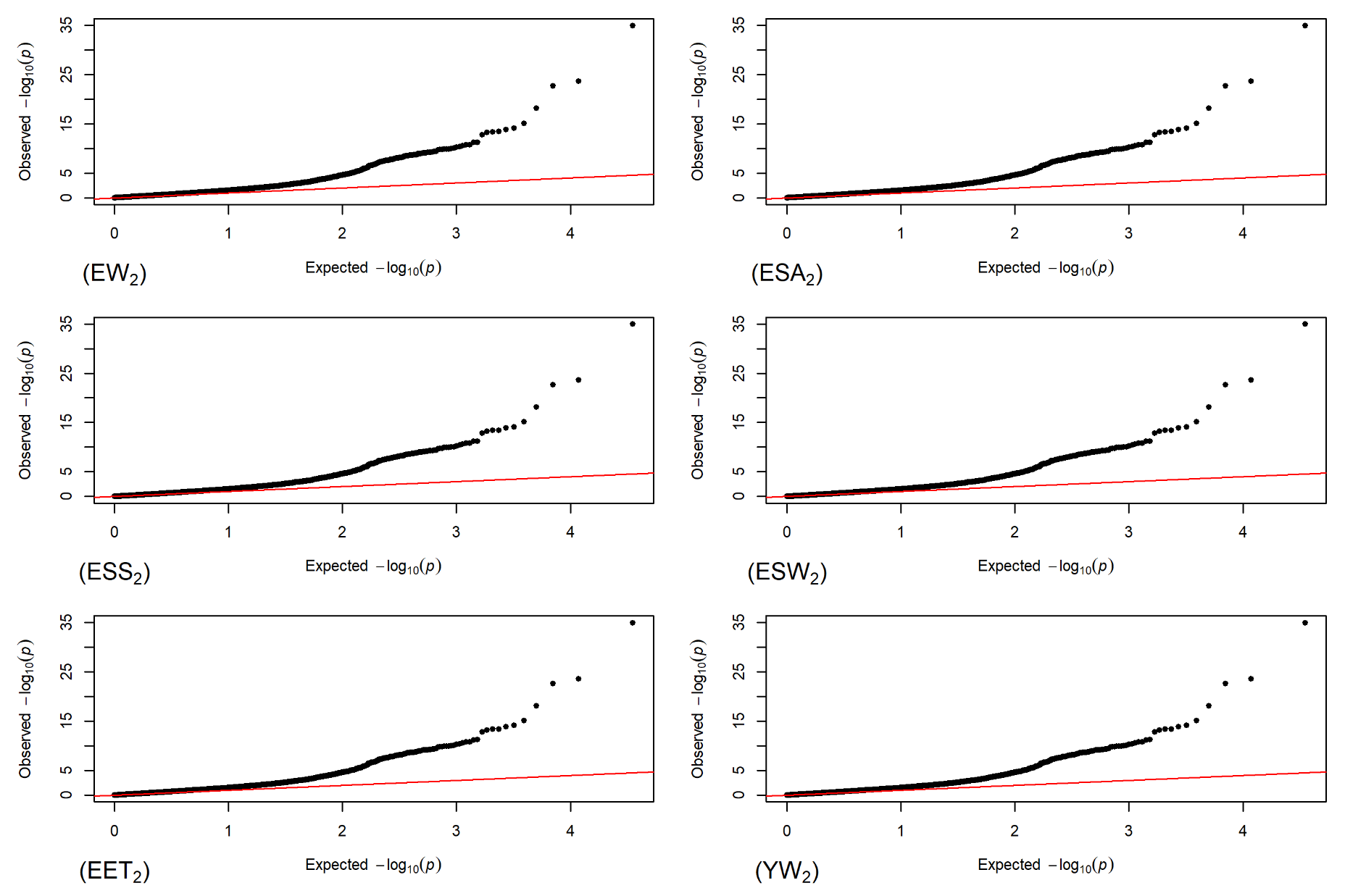


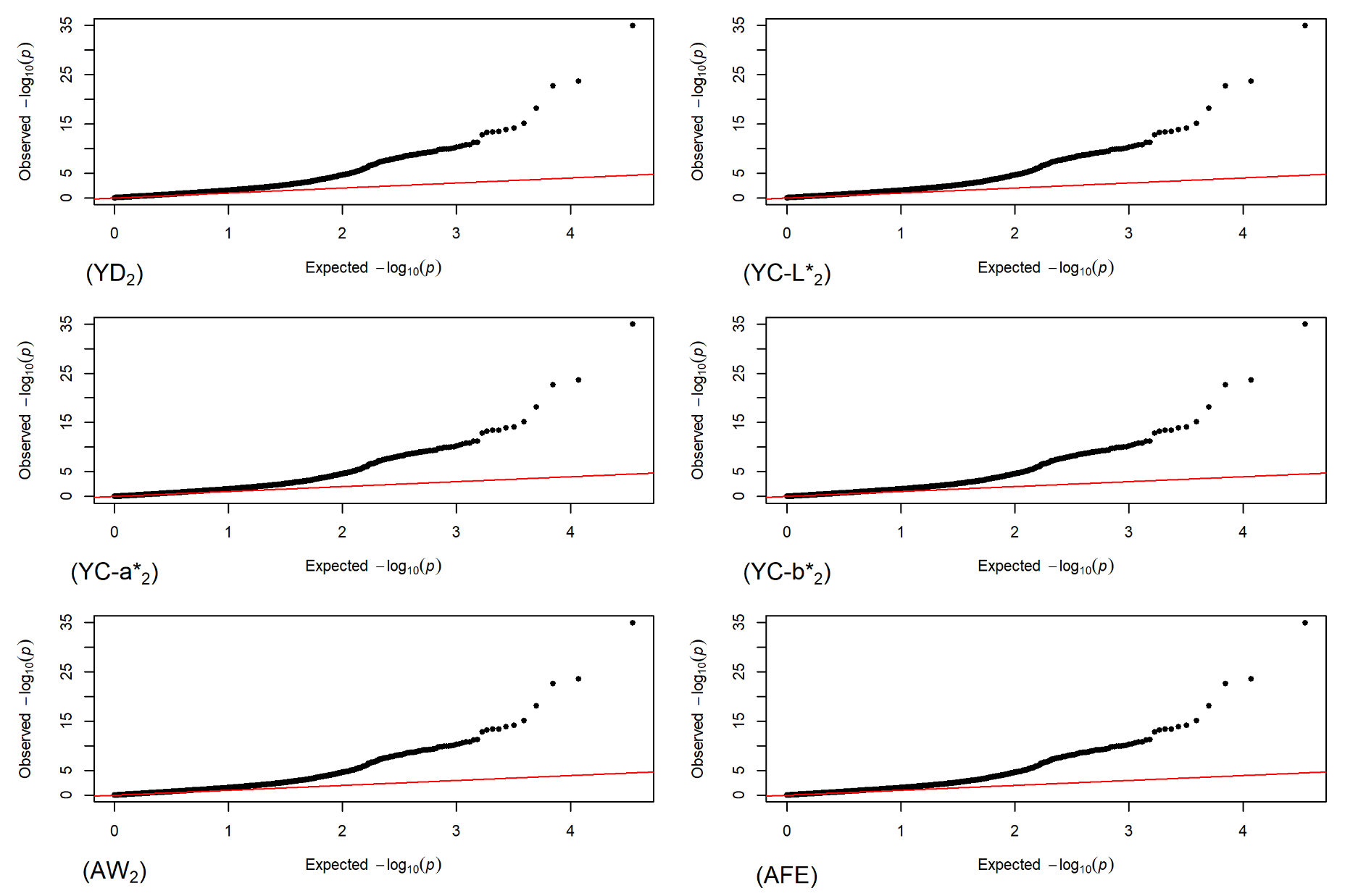


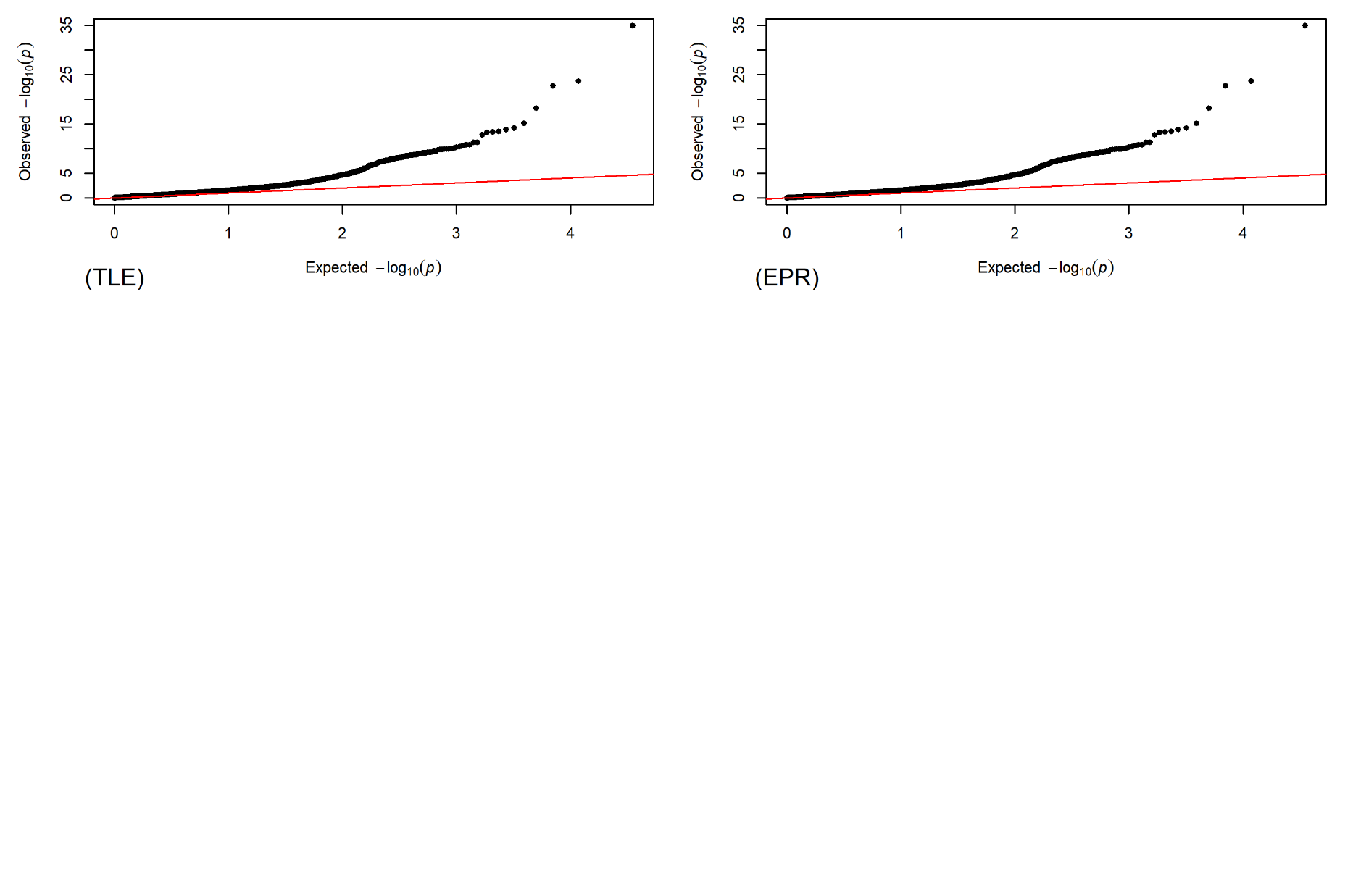


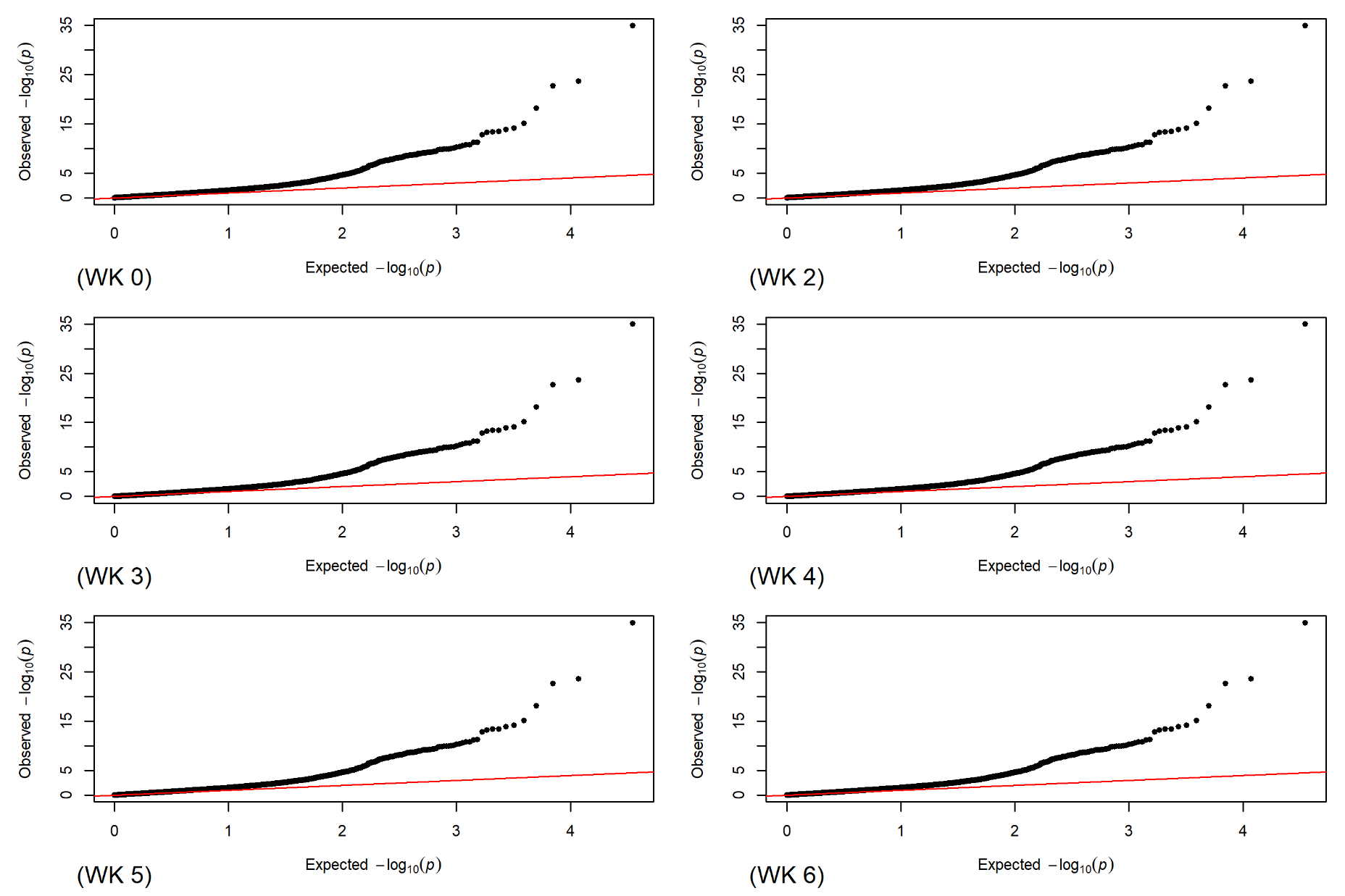


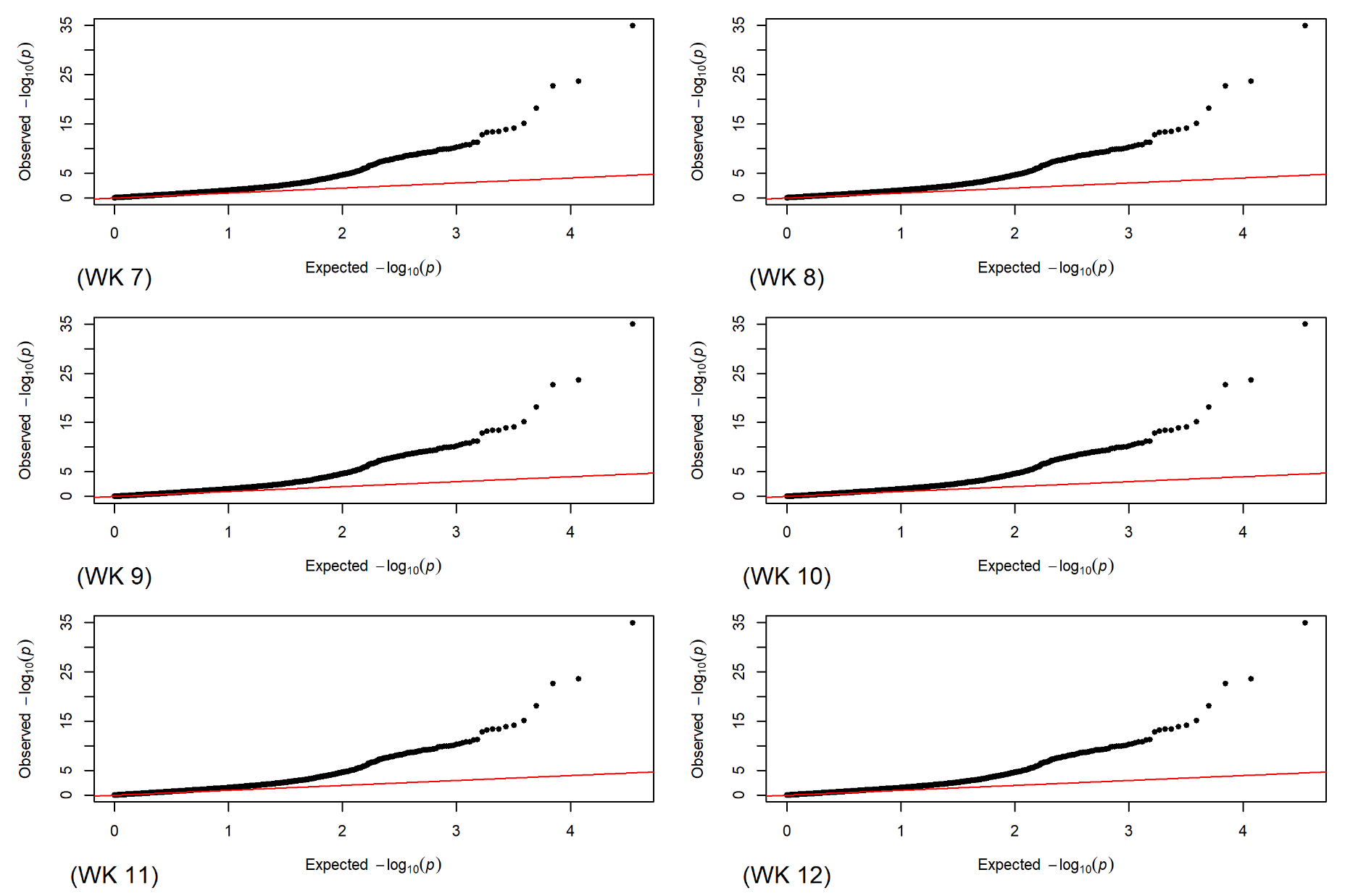


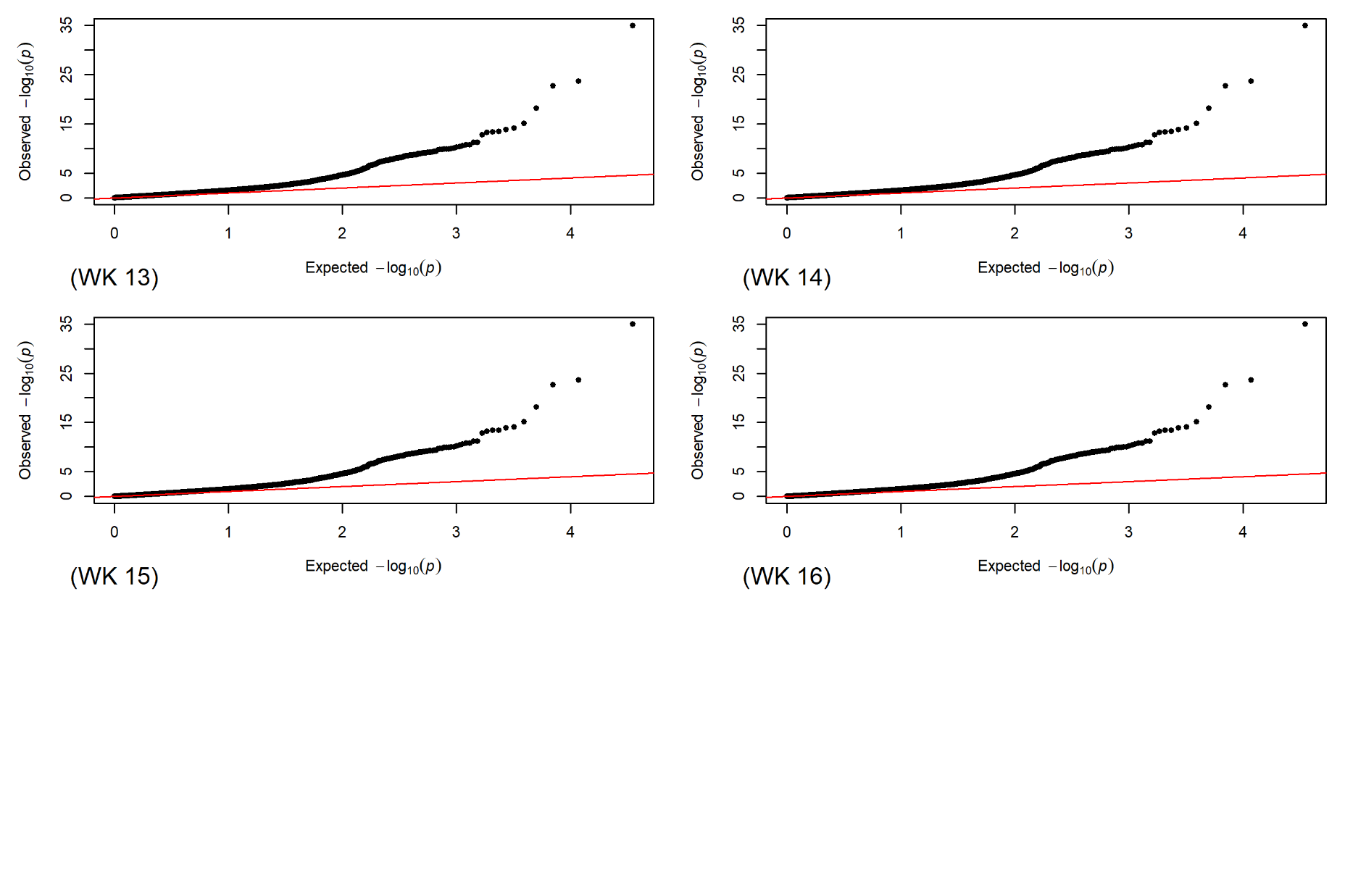


**Supplementary File 5.** The Manhattan and QQ plots for all traits (except for WK 1) using the MLM approach. In Manhattan figures, each dot represents an SNP. The figure illustrates the level of statistical significance (y-axis) as measured by the negative log of the corresponding *p*-value for each SNP. Each SNPs type is indicated by dots of different colours, which are arranged by chromosomal location (x-axis). The horizontal red line indicates the threshold of 5% Bonferroni genome-wide significance, and the blue line presents a genome-wide suggestive.
